# Incorporation of Bulky and Cationic Cyclam-Triazole Moieties into Marimastat Can Generate Potent MMP Inhibitory Activity without Inducing Cytotoxicity

**DOI:** 10.1002/open.201300014

**Published:** 2013-06-12

**Authors:** Mingfeng Yu, Ngee H Lim, Samantha Ellis, Hideaki Nagase, James A Triccas, Peter J Rutledge, Matthew H Todd

**Affiliations:** [a] School of Chemistry, The University of SydneySydney, NSW 2006 (Australia); [b] The Kennedy Institute of Rheumatology, University of Oxford65 Aspenlea Road, London W6 8 LH (United Kingdom); [c] Sydney Medical School, The University of SydneySydney, NSW 2006 (Australia)

**Keywords:** biological activity, click chemistry, marimastat, matrix metalloproteinase, metal complexes

## Abstract

The synthesis and matrix metalloproteinase (MMP) inhibitory activity of a cyclam–marimastat conjugate and its metal complexes are described. The conjugate, synthesized with a copper(I)-catalyzed Huisgen 1,3-dipolar cycloaddition (“click” reaction), contains two zinc-binding groups (ZBGs). The metal complexation behavior with copper(II) and zinc(II) was investigated using UV/Vis spectrophotometry and ^1^H NMR spectroscopy, respectively, demonstrating that the first equivalent of the metal ion was chelated by the cyclam-triazole moiety rather than the hydroxamic acid site. Thus, the corresponding mononuclear metal–cyclam complexes were successfully prepared with one equivalent of the metal salt. Both the cyclam–marimastat conjugate and its metal complexes exhibited slightly reduced potency against MMP-1, but essentially identical inhibitory activity against MMP-3. The conjugate and its metal complexes displayed little or no cytotoxicity, further supporting their potential suitability for imaging MMP localization and activity. To the best of our knowledge, this is the first report that describes the incorporation of metal complexes into an MMP inhibitor without influencing the preexisting ZBG, and the first report of the evaluation of structures containing more than one ZBG as MMP inhibitors.

## Introduction

Matrix metalloproteinases (MMPs) are zinc-containing endopeptidases capable of degrading extracellular matrix constituents.^1^ These enzymes play a pivotal role in a variety of physiological processes, including tissue remodeling, wound healing, embryonic development and angiogenesis.^2^ Overexpression of MMPs has been implicated in the progression of various pathologies, including cancer,^3^ arthritis^4^ and cardiovascular disease.^5^

The significance of these pathologies has led in turn to the development of a large number of synthetic MMP inhibitors (MMPIs).^6^ Most MMPs share a high degree of structural and functional similarity, which makes selective inhibition a major challenge.6a For many MMPIs, lack of target selectivity and/or undesired activity against other metalloproteinases trigger(s) dose-limiting side effects, typically musculoskeletal syndrome.3b

**Figure 1:**
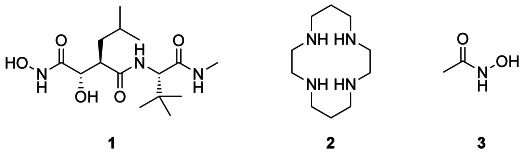
Structures of marimastat (**1**), cyclam (**2**) and acetohydroxamic acid (**3**).

Many MMPIs consist of a peptidomimetic backbone, which forms noncovalent interactions with the enzyme, and a zinc-binding group (ZBG) capable of chelating and ultimately inactivating the catalytic zinc(II) ion.^7^ Hydroxamates are monoanionic, bidentate chelators, and are commonly employed as ZBGs.^7^–^8^ Marimastat (**1**, Figure 1) is a well-studied hydroxamate-based MMPI, and was the first compound in this class to complete clinical trials as an anticancer drug.^9^ Although marimastat exhibits good oral bioavailability, development was eventually terminated because of severe side effects arising from a lack of selectivity.9a

Much recent research in the MMPI field has focused on the use of ZBGs other than hydroxamic acids as a way to increase selectivity.6a Cyclam **2** has recently been evaluated as a ZBG in this context and showed improved potency against MMPs relative to acetohydroxamic acid **3** (12-fold against MMP-1 and 19-fold against MMP-3).^10^ It has been proposed that the azamacrocycle acts by binding the active-site Zn^2+^, but this has not been experimentally demonstrated, and it is known that some MMPIs act without directly binding to the active-site metal.6a

**Figure 2:**
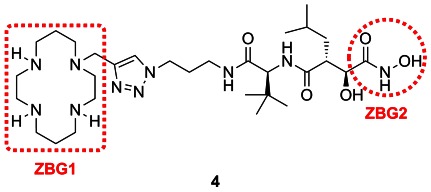
Structure of cyclam–marimastat conjugate **4**.

This report describes the synthesis and evaluation of the dual ZBG conjugate **4** (Figure 2). To the best of our knowledge, the plentiful literature on MMPIs does not include investigation of structures with more than one ZBG. Either or both the cyclam (ZBG1) and hydroxamic acid (ZBG2) moieties in **4** could act as ZBGs, and the interaction of compound **4** with MMPs and other proteins in vitro and in vivo was therefore of interest. Evaluation of the corresponding metal–cyclam complexes would afford insight into the effect of an appended metal–cyclam moiety (bulky and cationic) on the potency and selectivity of marimastat as an MMPI. There are surprisingly few previous reports of metal complexes being evaluated as MMPIs,^11]^ and none of the metal complexes previously studied also contain an additional, unmetallated ZBG. To the best of our knowledge, no MMPI has ever been synthesized with a remotely pendant metal complex. Complexes of this type, coordinated to an appropriate radionuclide, have potential as new tools for imaging MMP activity. Finally, construction of **4** and its metal–cyclam complexes extends our recent approach using copper(I)-catalyzed azide–alkyne cycloaddition (CuAAC; “click” reaction) to synthesize metal–cyclam complexes containing pendant biological ligands^12^ to complexes that include peptidomimetic protease inhibitors. Such methodology firstly shows the tolerance of the CuAAC reaction for more complex side chains, and secondly allows the assessment of the selectivity of metal binding when more than one chelating group is present.

## Results and Discussion

### Synthesis of the cyclam–marimastat conjugate 4

**Scheme 1:**
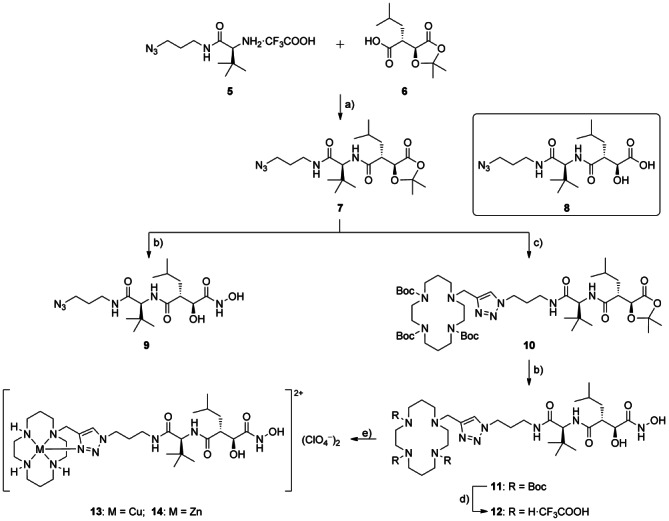
Synthesis of the cyclam–marimastat conjugate **12** and its metal complexes **13** and **14** as well as azide-capped marimastat **9**. *Reagents and conditions*: a) EDC⋅HCl (1.1 equiv), HOBt (1.1 equiv), DIPEA (3.0 equiv), DCM, RT, 2.5 h, 73 %; b) 50 % NH_2_OH/H_2_O, THF, reflux, 1 h, **9**: 81 %, **11**: 83 %; c) propargyl-tri-Boc cyclam (1.0 equiv), CuSO_4_⋅5 H_2_O (0.05 equiv), sodium ascorbate (0.10 equiv), *t*BuOH/H_2_O (1:1), RT, 1.5 h, 82 %; d) TFA/DCM/H_2_O (90:5:5), RT, 6 h, followed by RP-HPLC purification, 95 %; e) M(ClO_4_)_2_⋅6 H_2_O (M=Cu or Zn) (1.0 equiv), EtOH, reflux, 3 h, **13**: 63 %, **14**: 89 %.

Cyclam–marimastat conjugate **4** and its metal complexes were synthesized following a convergent strategy (Scheme 1). The two required precursors **5**^13^ and **6**13d, 14 were successfully prepared according to literature procedures (see the Supporting Information). It is known that a dioxolanone ring like that in **6** can be opened by hydrochloric acid to give an α-hydroxycarboxylic acid.14e Thus mixing the two precursors **5** and **6** carried the risk that residual trifluoroacetic acid (TFA) in the amine salt **5** could catalyze the same ring opening to give a mixture of the desired acetonide **7** and the unwanted α-hydroxycarboxylic acid analogue **8**. Coupling of amine trifluoroacetate **5** to carboxylic acid **6** was therefore effected by modifying the literature procedures:13d, 14e residual TFA in crude **5** was first neutralized with *N*,*N*-diisopropylethylamine (DIPEA) before the resulting material was added slowly to a solution of carboxylic acid **6** in anhydrous dichloromethane (DCM) in the presence of 1-ethyl-3-(3-dimethylaminopropyl)carbodiimide hydrochloride (EDC⋅HCl), hydroxybenzotriazole (HOBt) and DIPEA. In this way, the azide-capped acetonide **7** was obtained in 73 % yield from the coupling of **5** to **6**, a higher yield than those previously reported for coupling **6** or analogues to similar amine trifluoroacetates.13d, 14e, 15 The undesired α-hydroxycarboxylic acid **8** was isolated in ≤5 % yield. The ^1^H NMR spectrum of flash-column-purified **7** indicated that the sample contained less than 10 % of the unwanted diastereoisomers according to a comparison of peak integrals. These impurities were removed by recrystallization (ethyl acetate/hexane, 1:20; see the Supporting Information).

Literature reports suggested that hydroxamic acid **9,** formed by opening the dioxolanone ring of **7** with hydroxylamine, has considerable potential to bind metal ions.^7^, ^16^ Indeed yields for the copper(I)-catalyzed Huisgen 1,3-dipolar cycloaddition between alkynes and azides containing hydroxamic acids are generally very poor,^17^ suggesting that interaction of the hydroxamic acid with copper can hinder this reaction. As a result, the reaction between azide **7** and propargyl-tri-Boc cyclam (prepared as reported previously12a, c, 18) was carried out prior to ring opening of acetonide (Scheme 1). This cycloaddition was initially performed overnight (12–18 hours) to give poor and variable yields (24–52 %). Monitoring the reaction by TLC and LC-MS, it was found that the cycloaddition was complete after 1.5 hours, and that desired product **10** gradually underwent ring opening to the corresponding α-hydroxycarboxylic acid (revealed by appearance of a new peak at *m/z* 924.4). Therefore, the reaction was quenched after 1.5 hours, and the yield significantly improved to 82 %.

The dioxolanone ring of **10** was opened by direct nucleophilic attack of hydroxylamine^15^ to give the hydroxamic acid **11** in 83 % yield (Scheme 1). Removal of the Boc groups was carried out under previously optimized conditions (TFA/DCM/H_2_O, 90:5:5);12a the crude product was purified by reversed-phase HPLC (see the Supporting Information) to afford trifluoroacetate **12** in excellent yield (95 %). Isolation of the free amine from trifluoroacetate **12** was hampered by the exceptional solubility of **4** in water (bestowed by the combination of hydroxamic acid, hydroxyl group and three secondary amines). Therefore, trifluoroacetate **12** was directly used in subsequent metal complexation reactions.

### Metal complexation

The cyclam–marimastat conjugate **12** contains two ZBGs: the cyclam (ZBG1) and hydroxamic acid (ZBG2) moieties. Hence, complexation of this conjugate with one equivalent of a metal ion could give rise to four different binding modes: the metal ion could be exclusively chelated by (1) cyclam or (2) the hydroxamic acid, it could (3) interact in part with each ZBG, or (4) dynamically move from a kinetic interaction with one ZBG to a thermodynamic interaction with the other. Either the first or fourth binding mode is required to achieve the synthesis of the target metal–cyclam complexes **13** and **14** (Scheme 1), but it is of wider interest to establish the chronology of the **12**–metal interaction.

In order to determine the mode of binding between conjugate **12** and metal ions, copper(II) and zinc(II) were chosen for study using complementary analytical methods: UV/Vis spectrophotometry for copper(II) and ^1^H NMR spectroscopy for zinc(II). As a control, azide-capped marimastat **9** was synthesized (Scheme 1) to allow characterization of the interaction between the hydroxamic acid moiety and these metal ions.

**Figure 3:**
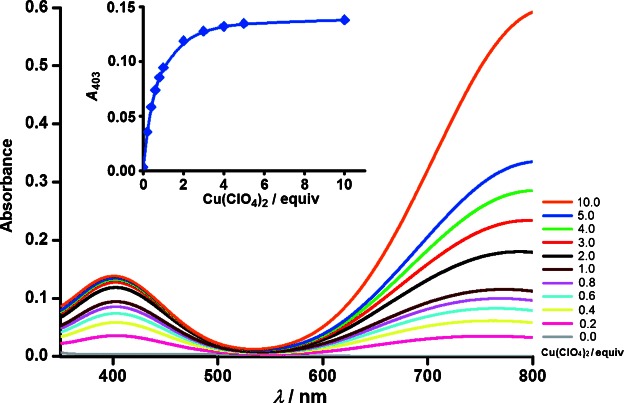
UV/Vis spectrophotometric titration of hydroxamic acid **9** (5 mm) with Cu(ClO_4_)_2_ (500 mm) at intervals of 5 min in CH_3_OH at 25 °C (inset: absorbance at 403 nm versus equivalents of Cu(ClO_4_)_2_ added).

Spectrophotometric titration of hydroxamic acid **9** with Cu(ClO_4_)_2_ in methanolic solution was performed (Figure 3) to obtain a *λ*_max_ value for the copper(II) complex of **9** as a reference for the titration of the cyclam–marimastat conjugate **12** with Cu(ClO_4_)_2_. The absorbance at 403 nm in the titration spectra increased when copper(II) salt was added up to ten equivalents, thus indicating chelation of Cu^2+^ by the hydroxamic acid moiety in **9**. A gradual decrease in the intervals of absorbance increase suggests that this complexation involves a weaker association than that typically observed for complexation of Cu^2+^ by *N*-functionalized cyclam derivatives.12a The absorbance apparent at 800 nm in Figure 3 is due to the metal salt, as the simple addition of Cu(ClO_4_)_2_ into methanol gave rise to this absorbance in the same manner.

**Figure 4:**
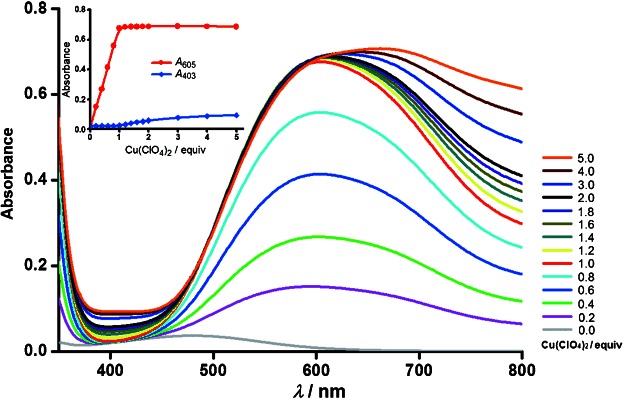
UV/Vis spectrophotometric titration of the cyclam–marimastat conjugate **12** (5 mm) with Cu(ClO_4_)_2_ (500 mm) at intervals of 5 min in CH_3_OH at 25 °C (inset: absorbances at 403 nm and 605 nm versus equivalents of Cu(ClO_4_)_2_ added).

Cyclam–marimastat conjugate **12** was titrated with Cu(ClO_4_)_2_ under the same conditions (Figure 4). An absorbance at 605 nm increased essentially linearly with the addition of Cu(ClO_4_)_2_, reaching a maximum upon addition of one equivalent of copper(II). During this time course (ca. 30 minutes), no increase of absorbance was observed elsewhere in the spectrum, including at ∼403 nm, implying that the first equivalent of copper(II) added interacted only with the cyclam site and not with the hydroxamic acid. Further addition of Cu(ClO_4_)_2_ up to five equivalents resulted in a continuous rise of the absorbance at ∼403 nm, with the increment of this rise progressively decreasing. As expected, the magnitude of this increase is similar to that observed in the case of hydroxamic acid **9**. Given that the typical *λ*_max_ values for copper(II) complexes of *N*-functionalized cyclam (550–625 nm)12a are significantly different to those for the copper(II)–hydroxamate complexes (∼403 nm, as seen for **9**), these UV/Vis titration profiles imply that a stoichiometric 1:1 complexation of the cyclam unit in **12** with Cu(ClO_4_)_2_ occurs in the first instance, followed by an interaction between the hydroxamic acid and the metal ion.

The different *λ*_max_ values of the copper(II) complexes of **9** and **12** manifested in clear differences between these solutions discernible to the naked eye: the former gave a pale green solution, while the latter appeared dark blue (see the Supporting Information).

**Figure 5:**
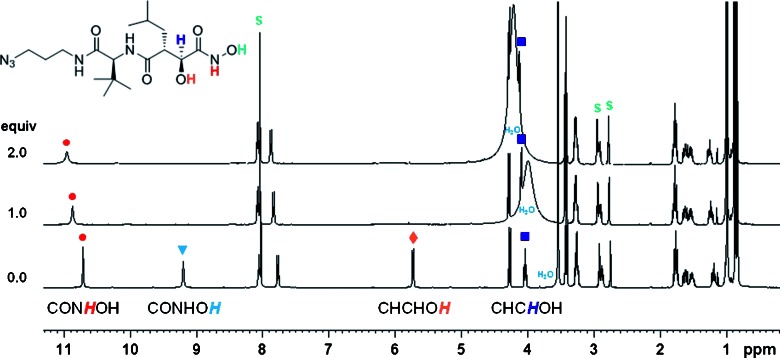
^1^H NMR spectroscopic titration of hydroxamic acid **9** (0.125 m) with Zn(ClO_4_)_2_ (1.00 m) at intervals of 15 min in [D_7_]DMF at 25 °C. S=nondeuterated solvent residual peaks.

**Figure 6:**
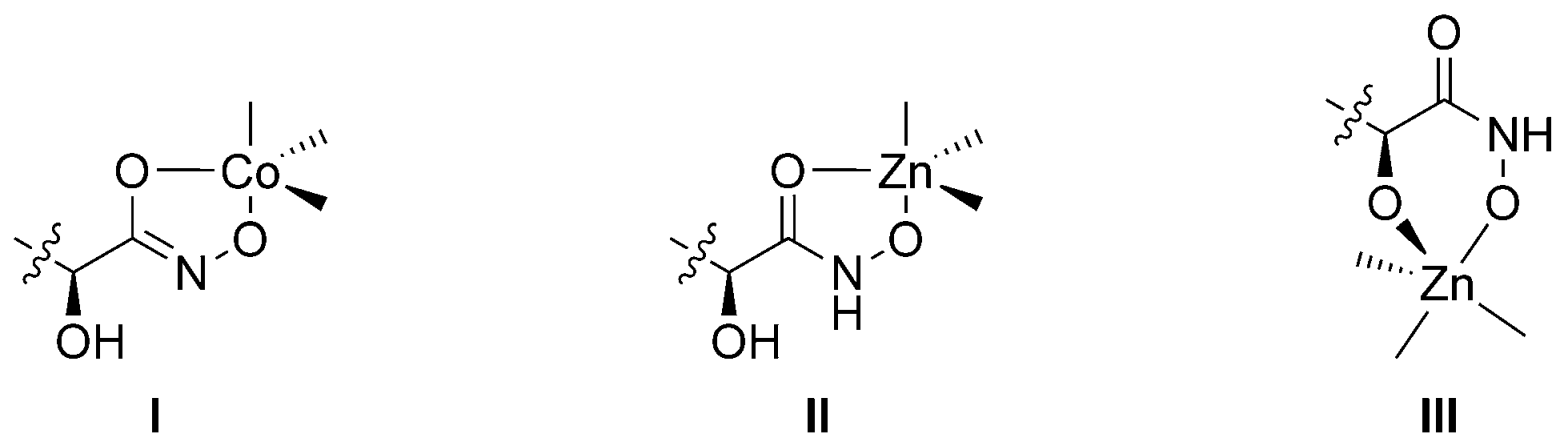
Previously reported (**I**),11a our expected (**II**) and apparent (**III**) modes of binding between α-hydroxy hydroxamic acid and a metal ion.

The corresponding titrations of **9** and **12** with Zn(ClO_4_)_2_ were monitored by ^1^H NMR spectroscopy. A deuterated solvent screen showed that all four proton signals of the α-hydroxy hydroxamic acid (C*H*(O*H*)CON*H*O*H*) in **9** were only observed when deuterated *N*,*N*-dimethylformamide ([D_7_]DMF) was used as solvent (Figure 5). These four proton signals were assigned by comparison with literature ^1^H NMR data for marimastat and α-dehydroxy marimastat analogues.9b, 13d, 14e The methine proton (CHC*H*OH) couples to both the adjacent methine and hydroxyl protons with a coincident coupling constant (6.8 Hz), giving rise to an apparent triplet at 4.04 ppm, and the hydroxyl proton (CHCHO*H*) signal splits into a doublet at 5.73 ppm. The addition of one equivalent of Zn(ClO_4_)_2_ resulted in (1) the disappearance of two hydroxyl proton signals due to metal-ion-induced deprotonation, (2) a downfield shift and broadening of the signals arising from the hydroxamic NH and residual H_2_O protons, and (3) a multiplicity change from an apparent triplet to a doublet for the methine proton (CHC*H*OH) signal, caused by deprotonation of the neighbouring hydroxyl group. Further addition of zinc(II) beyond one equivalent did not prompt any further substantial changes in the ^1^H NMR spectrum. These results imply that the hydroxyl group adjacent to the hydroxamic acid is involved in the binding of **9** to Zn^2+^, which, to the best of our knowledge, has not been previously reported (Figure 6).

**Figure 7:**
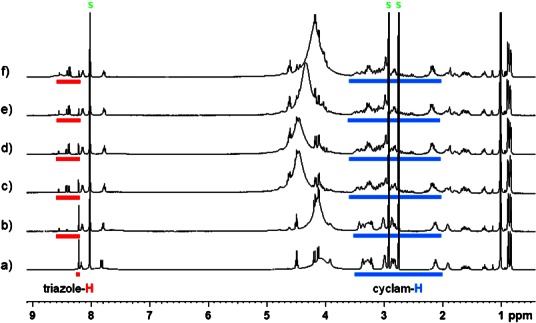
^1^H NMR spectroscopic titration of the cyclam–marimastat conjugate **12** (0.02 m) with Zn(ClO_4_)_2_ (1 m) in [D_7_]DMF. a) 0.0 equiv; b) 1.0 equiv, 25 °C, 5 min; c) 1.0 equiv, 80 °C, 5 min; d) 1.0 equiv, 80 °C, 1 h; e) 2.0 equiv, 80 °C, 1 h; f) 3.0 equiv, 80 °C, 1 h. S=nondeuterated solvent residual peaks.

It was expected that [D_7_]DMF could also be used for the titration of the cyclam–marimastat conjugate **12** with Zn(ClO_4_)_2_; however, the ^1^H NMR spectrum of **12** in the absence of metal salt surprisingly failed to give clear signals for any of the three α-hydroxy hydroxamic acid protons (CH(O*H*)CON*H*O*H*) (Figure 7 a). A further deuterated solvent screen tested deuterated chloroform (CDCl_3_), water (D_2_O), dimethyl sulfoxide ([D_6_]DMSO) and acetonitrile (CD_3_CN), but none of these solvents allowed clear visualization of these three protons (data not shown). Based on the UV/Vis spectrophotometric study of **12** with Cu^2+^, it was envisaged that Zn^2+^ was likely to be directly chelated by the cyclam moiety upon addition of one equivalent of the metal ion. As such, despite the obfuscated nature of the three α-hydroxy hydroxamic acid proton signals in [D_7_]DMF, the ^1^H NMR titration of **12** with Zn^2+^ was nonetheless performed in this solvent for consistency with the ^1^H NMR study with **9**. Attention focused on the signals arising from the cyclam-triazole moiety in the ^1^H NMR spectra (Figure 7). It was found that all well-defined proton signals of **12**, particularly in the regions corresponding to the cyclam and triazole protons, were split into multiplets upon addition of one equivalent of Zn^2+^ at 25 °C. The triazole singlet proton signal of **12** was split into six discrete singlets; these appeared poorly resolved when zinc(II) was first introduced, but were fully resolved after heating the sample at 80 °C for 1 hour. Comparing these changes with the ^1^H NMR spectra of previously reported zinc(II)-cyclam complexes12a suggests that complexation of the cyclam-triazole moiety within **12** with Zn^2+^ has occurred. Further changes to the cyclam- and triazole-derived signals were not observed when a total of three equivalents of zinc(II) were added. Taken together, these results suggest that the first equivalent of Zn^2+^ does indeed coordinate to the cyclam-triazole moiety.

Both UV/Vis spectrophotometric and ^1^H NMR spectroscopic studies clearly demonstrated that the first proposed binding mode dominates the complexation of cyclam–marimastat conjugate **12** with zinc(II) and copper(II) when only one equivalent of the metal ion is added. This complexation behaviour provided simple synthetic access to the target metal–cyclam complexes **13** and **14**. The complexation of trifluoroacetate **12** with one equivalent of copper(II) or zinc(II) perchlorate was performed in ethanol at reflux for 3 hours to ensure complete conversion, and the corresponding mononuclear metal–cyclam complexes **13** and **14** were obtained as blue and white powders in yields of 63 % and 89 %, respectively (Scheme 1). The constituent cations and anions of the metal complexes, that is, [M–ClO_4_]^+^ and ClO_4_^−^, were observed as characteristic signals in the high resolution mass spectra and IR spectra, respectively. Elemental analysis data showed that these metal–cyclam complexes contain up to three equivalents of water.

### Biological evaluation

#### MMP inhibition studies

**Figure 8:**
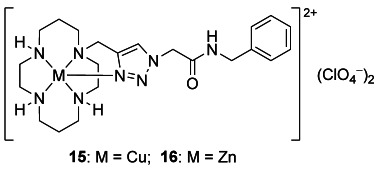
Structures of the two metal–cyclam-based control compounds that do not contain a hydroxamic acid.

The ability of the marimastat derivatives **7**–**9** and **11**–**14** to inhibit MMP-1 and MMP-3 was tested using the established assay with the fluorogenic substrate Mca-Pro-Leu-Gly-Leu-Dpa-Ala-Arg-NH_2_ (see the Supporting Information).^19^ Marimastat **1**, cyclam **2** and metal complexes **15** and **16** (Figure 8)12a were also assayed against these MMPs as controls. The data collected from these assays were fitted to the tight-binding inhibitor equation^20^ to obtain apparent inhibition constant (*K*_i_(app)) values (Table 1).

**Table 1:** *K*_i_(app) values [nm] for the marimastat-derived compounds against MMP-1 and MMP-3

Compd	MMP-1	MMP-3	Compd	MMP-1	MMP-3
**1**	2.1±0.5	12.3±1.0	**12**	18.8±1.3	12.8±1.1
**2**	nid^[a]^	nid^[a]^	**13**	17.9±1.0	34.1±1.4
**7**	6601±15	nid^[a]^	**14**	28.0±1.1	44.1±2.1
**8**	nid^[a]^	nid^[a]^	**15**	nid^[a]^	nid^[a]^
**9**	18.5±1.3	26.2±1.8	**16**	nid^[a]^	nid^[a]^
**11**	20.3±1.5	22.4±1.2			

[a] nid=no inhibition detected at 10 μm.

The cyclam–marimastat conjugate **12** exhibited slightly diminished efficacy (ca. 9-fold lower) against MMP-1 in comparison to marimastat **1**. However, the potency of this conjugate against MMP-3 is comparable to that of marimastat **1**. Metallation of the cyclam-triazole moiety resulted in only a slight reduction in MMP inhibitory activity (see data for **13** and **14**); protection of the cyclam site with three bulky Boc groups had surprisingly little effect, with derivative **11** retaining a high inhibitory activity against both MMPs tested. No MMP inhibition was detected for cyclam **2** at 10 μm. These results suggest that the bulky cyclam moieties (protonated, metallated, or Boc-protected) exert little influence on the ability of these marimastat conjugates to inhibit both metalloproteinases, and that the marimastat unit (ZBG2) beats the cyclam scorpionand ligand (ZBG1) as a ZBG at the MMP active site.

Installing an azido group at the end of the alkyl chain in marimastat (as in **9**) slightly reduces MMP inhibitory activity, whereas further protection of the hydroxamic acid (as acetonide **7**) or its replacement with a carboxylic acid (**8**) leads to a total loss of MMP inhibitory activity. Metal complexes **15** and **16**, which lack the hydroxamic acid altogether, show no ability to inhibit MMPs. These results underline the importance of the hydroxamic acid for the MMP inhibitory activity of marimastat derivatives. Moreover, these data point towards a mechanism of action for compounds **9** and **11**–**14** that involves hydroxamic acid binding to the MMP active site metal ion—rather than a cyclam–zinc(II) interaction.

#### Cytotoxicity

The cytotoxicity of the marimastat derivatives **7**–**9** and **11**–**14** was assayed against human acute monocytic leukemia cells (THP1) in triplicate in the range of 0.5–50 μm for seven days. Cell death/viability was measured using the resazurin reduction assay and calculating percentage fluorescence compared to a nontreated control. With the exception of the Boc-protected cyclam–marimastat conjugate **11**, which resulted in some toxicity at the highest concentration tested, the marimastat derivatives did not display any cytotoxicity (see the Supporting Information).

## Conclusions

The synthesis of the cyclam–marimastat conjugate **12** was accomplished using “click” chemistry. The metal complexation behavior of this conjugate with copper(II) and zinc(II) has been studied using UV/Vis spectrophotometry and ^1^H NMR spectroscopy, respectively, demonstrating that both copper(II) and zinc(II) ions are first chelated by the cyclam-triazole moiety rather than the hydroxamic acid site when one equivalent of the metal ion is added. Thus, the mononuclear metal–cyclam complexes **13** and **14** could be prepared; to the best of our knowledge, for the first time, metal complexes have been incorporated into an MMPI.

MMP inhibition studies show that appending a cyclam-triazole moiety to marimastat reduces inhibitory activity against MMP-1 by approximately one order of magnitude, but has little or no effect on potency against MMP-3. Neither conjugate **12** nor its metal complexes **13** and **14** exhibited significant cytotoxicity to mammalian cells. Our results also indicate that it is the hydroxamic acid group—and not the cyclam unit—of these cyclam–marimastat conjugates that is key to the zinc binding interaction at the MMP active site. Future work with simpler analogues of **4** containing only the hydroxamic acid and the azamacrocycle (i.e., just the two ZBGs) would allow a more detailed investigation of their relative metal binding effectiveness at the enzyme active site. Furthermore, the results reported here open the way to a new strategy for imaging MMP localization and activity, using conjugates of marimastat and other MMPIs with metal complexes.

## Experimental Section

See the Supporting Information for full experimental procedures and spectral data.

## Supplementary material

As a service to our authors and readers, this journal provides supporting information supplied by the authors. Such materials are peer reviewed and may be re-organized for online delivery, but are not copy-edited or typeset. Technical support issues arising from supporting information (other than missing files) should be addressed to the authors.
