# Supplementary material for: Incorporation of Bulky and Cationic Cyclam-Triazole Moieties into Marimastat Can Generate Potent MMP Inhibitory Activity without Inducing Cytotoxicity
Source: ChemistryOpen. 2013 Jun 12;2(3):99–105. doi: 10.1002/open.201300014 (PMC3703814; doi:10.1002/open.201300014)
Supplement: Supplementary file 1 [file open0002-0099-SD1.pdf]

ChemistryOPEN

Supporting Information

© 2013 The Authors. Published by Wiley-VCH Verlag GmbH & Co. KGaA, Weinheim

Incorporation of Bulky and Cationic Cyclam-Triazole Moieties into Marimastat Can Generate Potent MMP Inhibitory Activity without Inducing Cytotoxicity

Mingfeng Yu,[a] Ngee H. Lim,[b] Samantha Ellis,[c] Hideaki Nagase,[b] James A. Triccas,[c] Peter J. Rutledge,*[a] and Matthew H. Todd*[a]

open_201300014_sm_miscellaneous_information.pdf

1. General Materials....S2

2. Instrumentation and Methods....S3

3. Synthesis of Precursors **5** and **6**....S5

4. Synthesis of Marimastat Derivatives **9** and **12-14**....S12

5. ^{1}H NMR Analysis for Recrystallization of Azide-Capped Marimastat **7**....S18

6. RP-HPLC Purification of the Cyclam-Marimastat Conjugate **12**....S19

7. Distinct Colours Observed for Metal Complexation Titrations....S20

8. Cell Viability Assay....S21

9. MMP Inhibition Assay....S22

10. References....S23

11. ^{1}H & ^{13}C NMR Spectra for Novel Compounds....S24

1. General Materials

All reactions were carried out with continuous magnetic stirring in ordinary glassware. Heating of reactions was conducted with a paraffin oil bath; cooling of reactions was achieved using an ice bath (0 °C) or an ethanol bath with Flexi-Cool® FC100D11 immersion cooler (-30 °C). All reagents and solvents were purchased from Sigma-Aldrich, Alfa Acer, Merck, Mimotopes, GL Biochem or Ajax Finechem. Reagents were used as received unless otherwise specified. Hexane and ethyl acetate were distilled before use. Dichloromethane and ethanol were distilled over calcium hydride and stored over activated 4 Å molecular sieves. Tetrahydrofuran was distilled over sodium wire/benzophenone. Diethyl ether was collected freshly from a PureSolv MD 7 solvent purification system having been passed through an anhydrous alumina column.

2. Instrumentation and Methods

^{1}H and ^{13}C NMR spectra were recorded at 300 K on a Bruker AVANCE 200 spectrometer (^{1}H at 200.13 MHz and ^{13}C at 50.32 MHz), a Bruker AVANCE 300 spectrometer (^{1}H at 300.13 MHz and ^{13}C at 75.47 MHz) or a Bruker DRX 400 spectrometer (^{1}H at 400.13 MHz and ^{13}C at 100.61 MHz). ^{1}H and ^{13}C NMR spectra are referenced to ^{1}H signals of residual nondeuterated solvents (or tetramethylsilane) and ^{13}C signals of the deuterated solvents respectively. ^{1}H NMR signals are reported with chemical shift values δ (ppm), multiplicity (s = singlet, d = doublet, t = triplet, q = quartet, dt = doublet of triplets, m = multiplet and br = broad), relative integral, coupling constants J (Hz) and assignments. Infrared spectra were recorded on a Bruker Alpha FT-IR spectrometer. UV-Vis spectra were recorded on a Varian Cary 4000 or Varian Cary 1E UV-visible spectrophotometer. Temperature was controlled by a Varian Cary PCB water peltier system. Low resolution and high resolution mass spectra were recorded on a Finnigan LCQ mass spectrometer and a Bruker 7T Fourier Transform Ion Cyclotron Resonance (FT-ICR) Mass Spectrometer respectively. Ionization of all samples was carried out using electrospray ionization (ESI) or atmospheric pressure chemical ionization (APCI). Optical rotation α was measured on a PerkinElmer 341 polarimeter with a sodium lamp in a semi-micro fused silica polarimeter cell (length: 100 mm, capacity: 3.0 mL) at 589 nm and 20 °C using spectroscopic grade solvents. Temperature was controlled by a Julabo F12-ED refrigerated/heating circulator connected directly to the polarimeter cell. Melting points were determined on an OptiMelt 100 automated melting point apparatus and are uncorrected. Elemental analyses were carried out by the Campbell Microanalytical Laboratory (University of Otago, New Zealand) on a Carlo Erba EA 1108 Elemental Analyser. Analytic reverse phase high performance liquid chromatography (RP-HPLC) was carried out on a Waters 2695 separations module with a Waters 2996 photodiode array detector and an Alliance series column heater. A Waters SunFire™ C18 column (5 µm, 2.1 × 150 mm) was used at 30 °C at a flow rate of 0.2 mL/min. Preparative RP-HPLC was carried out on a Waters 600 controller with a Waters 600 pump and a 2998 photodiode array detector. A Waters SunFire™ C18 OBD™ column (5 µm, 19 × 150 mm) was used at a flow rate of 7 mL/min. Mobile phases of 0.1% TFA in Milli-Q water (solvent A) and 0.1% TFA in acetonitrile (solvent B) in different ratios was used in both analytic and preparative RP-HPLC. The fractions from preparative RP-

HPLC were lyophilized using a Labconco FreeZone 6 liter console freeze dry system. Data acquired from both analytic and preparative RP-HPLC were processed using Waters Empower 2 software. Liquid chromatography mass spectrometry (LCMS) was performed on a Thermo Separation Products: Spectra System consisting of a P400 pump and a UV6000LP photodiode array detector coupled to a Thermoquest Finnigan LCQ Deca mass spectrometer (ESI). A Phenomenex Jupiter C18 column (5 µm, 2.1 × 150 mm) was eluted at a flow rate of 0.2 mL/min with a mobile phase of 0.1% formic acid in Milli-Q water and 0.1% formic acid in acetonitrile. Analytical thin layer chromatography (TLC) was performed on Merck silica gel 60 F_{254} pre-coated aluminium plates (0.2 mm) and visualized under UV light (254 nm), followed by staining with ninhydrin or phosphomolybdic acid (PMA). Flash column chromatography was carried out using Merck silica gel 60 (0.040-0.063 mm).

3. Synthesis of Precursors 5 and 6

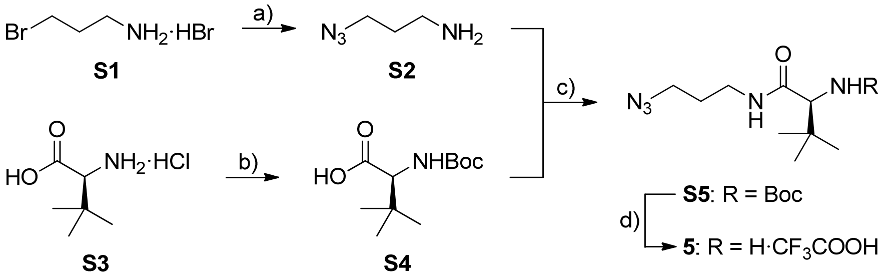

Scheme S1. Synthesis of precursor **5**. Reagents and conditions: (a) NaN_3, H_2O, reflux, 16 h, 88%; (b) Boc_2O, NaOH, *t*BuOH/H_2O (1:1), rt, o/n, 96%; (c) EDC·HCl, HOBt, DIPEA, DCM, rt, o/n, 89%; (d) TFA/DCM/H_2O (20:5:1), rt, 2 h, 98%.

3-Azidopropan-1-amine (S2).[1]

To a solution of 3-bromopropan-1-amine hydrobromide (**S1**, 14.2 g, 64.9 mmol) in H_2O (45 mL) was added dropwise a solution of sodium azide (14.1 g, 217 mmol) in H_2O. The reaction mixture was heated at reflux for 16 h and concentrated to one-third its original volume under reduced pressure. To the ice-cooled residue was added Et_2O (225 mL), followed by KOH pellets (18.0 g, 321 mmol) portionwise keeping the temperature below 10 °C. The organic layer was separated, and the aqueous layer was extracted with Et_2O (2 × 135 mL). The combined organic layers were dried over K_2CO_3 and concentrated under reduced pressure and *in vacuo* to give **S2** as a pale yellow oil (5.73 g, 88%). **IR** ν_{max}/cm^{-1} 3368, 3294, 2938, 2871, 2089, 1596, 1458, 1256, 846. **^{1}H NMR** (300 MHz, $CDCl_3$) δ 1.22 (s, 2H, NH_2), 1.67-1.77 (m, 2H, $CH_2CH_2CH_2$), 2.79 (t, 2H, *J* 6.6, NH_2CH_2), 3.37 (t, 2H, *J* 6.6, CH_2N_3). **^{13}C NMR** (75 MHz, $CDCl_3$) δ 32.2, 39.0, 48.8. **MS** (APCI) *m/z* 100.5 ([M+H]$^+$, 100%). The spectroscopic data were in agreement with those in the literature.[1]

(*S*)-2-((*tert*-Butoxycarbonyl)amino)-3,3-dimethylbutanoic acid (S4).[2]

To a solution of L-*tert*-leucine (**S3**, 4.58 g, 34.9 mmol) and NaOH (1.54 g, 38.5 mmol) in *t*BuOH/H_2O (50 mL/50 mL) was added di-*tert*-butyl dicarbonate (8.40 g, 38.5 mmol). The reaction mixture was stirred at room temperature overnight and washed with EtOAc (3 × 100 mL), and the combined organic layers were back-extracted with saturated $NaHCO_3$ (6 × 50 mL). The combined aqueous phases were taken to pH 1 with

6 M HCl and extracted with EtOAc (5 × 50 mL). The combined organic extracts were dried over Na_2SO_4 and concentrated under reduced pressure to give **S4** as a white solid (7.78 g, 96%). ***R*$_F$** (EtOAc:hexane = 1:1) 0.63. **m.p.** 117-118 °C (lit.[2b] **m.p.** 122 °C). **$[\alpha]_D^{20}$** +6.1 (*c* 0.61, EtOAc) (lit.[2b] **$[\alpha]_D^{24}$** +5.8 ± 0.1 (*c* 0.6, EtOAc)). **IR** ν_{max}/cm^{-1} 3443, 3326, 3101, 2971, 2622, 2530, 1710, 1508, 1401, 1371, 1331, 1233, 1162, 1060, 1013, 759. **^1H NMR** (300 MHz, $CDCl_3$) (Compound **S4** exists as two rotamers in approximately 7:4 ratio. Major rotamer is designated as $^{\#}$ and minor rotamer is designated as *.) δ 1.02 (s, 9H, $CHC(CH_3)_3$), 1.45 (s, 9H, $COOC(CH_3)_3$), 3.88* (d, 1H, *J* 5.4, C*H*COOH), 4.14$^{\#}$ (d, 1H, *J* 9.3, C*H*COOH), 5.21$^{\#}$ (d, 1H, *J* 9.0, CONH), 6.48* (d, 1H, *J* 4.5, CONH), 11.12 (br s, 1H, COOH). **^{13}C NMR** (75 MHz, $CDCl_3$) δ 26.6, 28.4, 34.0*, 34.6$^{\#}$, 61.7$^{\#}$, 63.8*, 80.1$^{\#}$, 81.8*, 155.8$^{\#}$, 157.1*, 176.5 (four carbon signals overlapping or obscured). **MS** (ESI) *m/z* 229.7 ($[M-H]^-$, 100%). The spectroscopic data were in agreement with those in the literature.[2]

(*S*)-*tert*-Butyl (1-((3-azidopropyl)amino)-3,3-dimethyl-1-oxobutan-2-yl)carbamate (S5).

To a solution of compound **S2** (5.05 g, 50.4 mmol) and compound **S4** (7.78 g, 33.6 mmol) in anhydrous DCM (140 mL) were added EDC·HCl (7.09 g, 37.0 mmol), HOBt (5.00 g, 37.0 mmol) and DIPEA (35.0 mL, 201 mmol). The reaction mixture was stirred at room temperature under N_2 overnight and washed with 10% citric acid (3 × 50 mL), 5% $NaHCO_3$ (3 × 50 mL) and brine (1 × 50 mL). The organic phase was dried over $MgSO_4$, filtered and concentrated under reduced pressure. The residue was purified by flash column chromatography (silica gel, DCM ramping to DCM:EtOAc = 85:15) to give **S5** as a white solid (9.36 g, 89%). ***R*$_F$** (DCM:EtOAc = 4:1) 0.66. **m.p.** 92-93 °C. **$[\alpha]_D^{20}$** +5.7 (*c* 1.0, $CHCl_3$). **IR** ν_{max}/cm^{-1} 3440, 3312, 3084, 2967, 2876, 2095, 1651, 1507, 1365, 1312, 1241, 1165, 1070, 1009, 966, 911, 861, 780, 734, 643, 558. **^1H NMR** (300 MHz, $CDCl_3$) δ 1.01 (s, 9H, $CHC(CH_3)_3$), 1.44 (s, 9H, $COOC(CH_3)_3$), 1.70-1.87 (m, 2H, $CH_2CH_2CH_2$), 3.22-3.43 (m, 2H, $CONHCH_2$), 3.34 (t, 2H, *J* 6.6, CH_2N_3), 3.95 (d, 1H, *J* 9.3, C*H*CONH), 5.53 (d, 1H, *J* 9.3, CON*H*CH), 7.14 (br s, 1H, CON*H*CH_2). **^{13}C NMR** (75 MHz, $CDCl_3$) δ 26.6, 28.3, 28.9, 34.3, 36.8, 49.2, 62.3, 79.5, 156.1, 171.3 (four carbon signals overlapping or obscured). **MS** (ESI) *m/z* 335.7 ($[M+Na]^+$, 100%), 648.7 ($[2M+Na]^+$, 21%). **HRMS** (ESI) 336.20037 ($[M+Na]^+$); calcd. for $C_{14}H_{27}N_5NaO_3$ ($[M+Na]^+$) 336.20061.

(*S*)-1-((3-Azidopropyl)amino)-3,3-dimethyl-1-oxobutan-2-aminium 2,2,2-trifluoroacetate (5).

Compound **S5** (157 mg, 0.501 mmol) was deprotected in a mixture of DCM/TFA/H_2O (40 mL/10 mL/2 mL) at room temperature overnight. The reaction mixture was concentrated under reduced pressure, and the residue was lyophilized to give **5** as a pale yellow glue (161 mg, 98%). **$[\alpha]_D^{20}$** +47.7 (*c* 1.0, CH_3CN). **IR** ν_{max}/cm^{-1} 3281, 3089, 2966, 2099, 1664, 1526, 1477, 1435, 1378, 1260, 1185, 1132, 1021, 934, 837, 799, 719. **^{1}H NMR** (300 MHz, D_2O) δ 0.95 (s, 9H, $C(CH_3)_3$), 1.64-1.74 (m, 2H, $CH_2CH_2CH_2$), 3.10-3.32 (m, 2H, $CONHCH_2$), 3.26 (t, 2H, *J* 6.6, CH_2N_3), 3.55 (s, 1H, C*H*CONH) (three ammonium proton signals (NH_3^+) and one amide proton signal (CONH) not observed due to H/D exchange). **^{13}C NMR** (75 MHz, D_2O) δ 25.9, 28.0, 33.1, 37.2, 49.1, 62.3, 116.5 (q, $J_{C\text{-}F}$ 292.5, CF_3), 162.4 (q, $J_{C\text{-}F}$ 37.5, CF_3*C*OOH), 168.5 (two carbon signals overlapping or obscured). **MS** (ESI) *m/z* 213.6 ([M-TFA+H]$^+$, 100%), 426.5 ([2(M-TFA)+H]$^+$, 9%). **HRMS** (ESI) 427.32454 ([2(M-TFA)+H]$^+$); calcd. for $C_{18}H_{39}N_{10}O_2$ ([2(M-TFA)+H]$^+$) 427.32520.

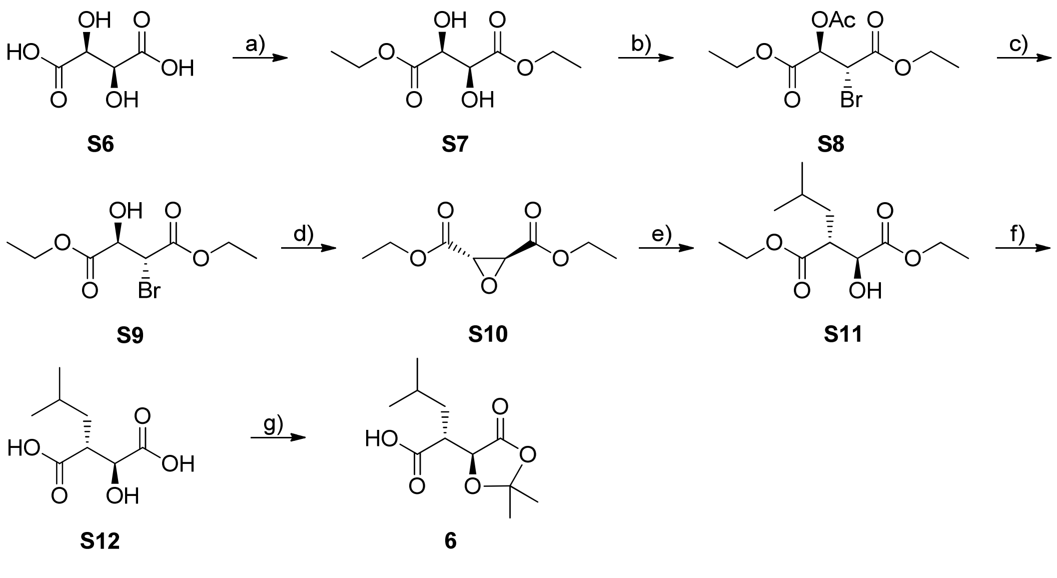

Scheme S2. Synthesis of precursor **6**. Reagents and conditions: (a) $SOCl_2$, EtOH, rt, 48 h, 100%; (b) 33% HBr/AcOH, 0 °C to rt, 16 h, 92%; (c) 33% HBr/AcOH, EtOH, reflux, 4.5 h, 91%; (d) DBU, Et_2O, 0 °C, 2 h, 86% or Na, EtOH, 0 °C to rt, 1 h, 61%; (e) isobutylmagnesium bromide, CuCN, Et_2O, -30 °C, 1.5 h, 46%; (f) NaOH, 1,4-dioxane/H_2O, rt, 19 h, 90%; (g) 2,2-dimethoxypropane, TsOH, rt, 2.5 h, 85%.

(2*S*,3*S*)-Diethyl 2,3-dihydroxysuccinate (*S*7).[3]

To a solution of D-tartaric acid (**S6**, 29.0 g, 0.193 mol) in anhydrous EtOH (290 mL) was added dropwise thionyl chloride (31.2 mL, 0.430 mol). The mixture was stirred at room temperature for 48 h and concentrated under reduced pressure. The residue was diluted with DCM (60 mL) and washed with saturated $NaHCO_3$ (40 mL). The aqueous layer was back-extracted with DCM (2 × 20 mL). The combined organic phases were washed with brine (40 mL), dried over Na_2SO_4 and concentrated under reduced pressure to give **S7** as a colourless oil (39.8 g, 100%). $[\alpha]_D^{20}$ -26.0 (*c* 0.2, H_2O) (lit.[3] $[\alpha]_D^{20}$ -25.0 (*c* 0.2, H_2O)). **IR** ν_{max}/cm^{-1} 3473, 2984, 2941, 2911, 2879, 1732, 1634, 1470, 1448, 1391, 1370, 1223, 1126, 1083, 1016, 862, 730, 708, 596. **^{1}H NMR** (300 MHz, $CDCl_3$) δ 1.32 (t, 6H, *J* 7.2, 2 × $COOCH_2CH_3$), 3.73 (br s, 2H, 2 × OH), 4.30 (q, 4H, *J* 7.2, 2 × $COOCH_2CH_3$), 4.56 (s, 2H, 2 × $CHCOOCH_2CH_3$). **^{13}C NMR** (75 MHz, $CDCl_3$) δ 13.9, 62.1, 72.2, 171.5 (four carbon signals overlapping or obscured). **MS** (ESI) *m/z* 228.7 ($[M+Na]^+$, 100%), 434.6 ($[2M+Na]^+$, 90%). The spectroscopic data were in agreement with those in the literature.[3]

(2*R*,3*R*) Diethyl 2-acetoxy-3-bromosuccinate (S8).[4]

To ice-cooled **S7** (20.0 g, 97.0 mmol) was added dropwise a solution of HBr in AcOH (33%, 60.0 mL) over 30 min. The mixture was allowed to warm to room temperature, stirred for 16 h and poured onto crushed ice (85 g) which was allowed to melt. The aqueous solution was extracted with Et_2O (4 × 100 mL). The combined organic layers were washed with H_2O (3 × 40 mL) and brine (30 mL), dried over $MgSO_4$ and concentrated under reduced pressure. The residue was purified by flash column chromatography (silica gel, EtOAc:hexane = 5:95 ramping to 10:90) to give **S8** as a colourless oil (27.7 g, 92%). R_F (EtOAc:hexane = 1:4) 0.40. $[\alpha]_D^{20}$ +6.5 (*c* 1.0, $CHCl_3$). **IR** ν_{max}/cm^{-1} 2984, 2941, 2906, 1740, 1468, 1446, 1372, 1271, 1205, 1159, 1096, 1021, 859. **^{1}H NMR** (200 MHz, $CDCl_3$) δ 1.31 (t, 6H, *J* 7.2, 2 × $COOCH_2CH_3$), 2.17 (s, 3H, $COCH_3$), 4.26 (q, 2H, *J* 7.2, $COOCH_2CH_3$), 4.28 (q, 2H, *J* 7.2, $COOCH_2CH_3$), 4.79 (d, 1H, *J* 5.4, $CHCOOCH_2CH_3$), 5.59 (d, 1H, *J* 5.4, $CHCOOCH_2CH_3$). **^{13}C NMR** (75 MHz, $CDCl_3$) δ 14.0, 14.1, 20.4, 43.9, 62.3, 63.0, 72.8, 165.7, 166.3, 169.4. **MS** (ESI) *m/z* 332.6 ($[M(^{79}Br)+Na]^+$, 100%), 334.5 ($[M(^{81}Br)+Na]^+$, 94%). The spectroscopic data were in agreement with those in the literature.[4]

(2*R*,3*R*)-Diethyl 2-bromo-3-hydroxysuccinate (S9).[4]

To a solution of compound **S8** (24.0 g, 77.4 mmol) in EtOH (250 mL) was added dropwise a solution of HBr in AcOH (33%, 8.20 mL). The mixture was heated at reflux for 4.5 h and concentrated under reduced pressure. The residue was purified by flash column chromatography (silica gel, EtOAc:hexane = 1:4) to give **S9** as a colourless oil (18.9 g, 91%). R_F (EtOAc:hexane = 1:1) 0.82. $[\alpha]_D^{20}$ +29.1 (*c* 1.0, $CHCl_3$) (lit.[4a] $[\alpha]_D^{23}$ +30.7 (neat)). **IR** ν_{max}/cm^{-1} 3476, 2984, 2940, 2908, 1729, 1468, 1447, 1392, 1370, 1261, 1214, 1154, 1099, 1019, 859. **^{1}H NMR** (300 MHz, $CDCl_3$) δ 1.31 (t, 6H, *J* 7.2, 2 × $COOCH_2CH_3$), 3.79 (d, 1H, *J* 6.9, OH), 4.22-4.35 (m, 2H, $COOCH_2CH_3$), 4.27 (q, 2H, *J* 7.2, $COOCH_2CH_3$), 4.65-4.73 (m, 2H, 2 × $CHCOOCH_2CH_3$). **^{13}C NMR** (75 MHz, $CDCl_3$) δ 13.8, 14.0, 47.5, 62.4, 62.7, 72.5, 166.7, 170.2. **MS** (ESI) *m/z* 290.6 ([M(^{79}Br)+Na]$^+$, 90%), 292.5 ([M(^{81}Br)+Na]$^+$, 100%). The spectroscopic data were in agreement with those in the literature.[4]

(2*S*,3*S*)-Diethyl oxirane-2,3-dicarboxylate (S10).

EtONa Method:[4a] To anhydrous EtOH (3 mL) was added Na (100 mg, 4.35 mmol), and the resulting solution went clear over the course of 15 min and was cooled on an ice bath. A solution of compound **S9** (1.00 g, 3.72 mmol) in anhydrous EtOH (3 mL) was added dropwise. The mixture was stirred at room temperature for 1 h, neutralized with AcOH and concentrated under reduced pressure. The residue was diluted with H_2O (10 mL) and extracted with Et_2O (3 × 10 mL). The combined organic layers were washed with H_2O (10 mL) and brine (10 mL), dried over $MgSO_4$ and concentrated under reduced pressure. The residue was purified by flash column chromatography (silica gel, EtOAc:hexane = 5:95) to give **S10** as a colourless oil (428 mg, 61%).

DBU method:[4b] To an ice-cooled solution of compound **S9** (17.9 g, 66.5 mmol) in anhydrous Et_2O (80 mL) was added dropwise a solution of DBU (13.0 mL, 86.9 mmol) in anhydrous Et_2O (40 mL) over 1.5 h. The reaction mixture was stirred for a further 30 min, and H_2O (12 mL) was added. The resulting mixture was washed with 1 M $KHSO_4$ (3 × 40 mL) and H_2O (2 × 40 mL), and concentrated under reduced pressure to give **S10** as a colourless oil (10.8 g, 86%).

***R*~F~** (EtOAc:hexane = 1:4) 0.40. **[α]$_D^{20}$** +111.0 (*c* 1.416, Et_2O) (lit.[4a] **[α]$_D^{23}$** +105.49 (*c* 1.413, Et_2O)). **IR** ν_{max}/cm^{-1} 2985, 2940, 2910, 1744, 1469, 1448, 1394, 1371, 1328, 1278, 1248, 1231, 1197, 1095, 1027, 900. **^{1}H NMR** (300 MHz, $CDCl_3$) δ 1.32 (t, 6H, *J* 7.2, 2 × $COOCH_2CH_3$), 3.66 (s, 2H, 2 × CH), 4.21-4.35 (m, 4H, 2 × $COOCH_2CH_3$). **^{13}C NMR** (75 MHz, $CDCl_3$) δ 13.8, 51.8, 61.9, 166.6 (four carbon signals overlapping or obscured). **MS** (ESI) *m/z* 398.8 ([2M+Na]$^+$, 100%), 586.9 ([3M+Na]$^+$, 15%), 774.6 ([4M+Na]$^+$, 50%). The spectroscopic data were in agreement with those in the literature.[4]

(2*S*,3*R*)-Diethyl 2-hydroxy-3-isobutylsuccinate (S11).[2b,5]

CuCN (4.53 g, 50.6 mmol) was suspended in anhydrous Et_2O (30 mL) under Ar and cooled to -30 °C. A 2.0 M solution of isobutylmagnesium bromide in Et_2O (23.0 mL, 46.0 mmol) was quickly added with vigorous stirring. After 30 min, a solution of compound **S10** (4.33 g, 23.0 mmol) in anhydrous Et_2O (20 mL) was added dropwise to the suspension, and the mixture was stirred at -30 °C for 1.5 h. The reaction was quenched with saturated NH_4Cl and filtered through a pad of Celite. The phases were separated, and the aqueous phase was extracted with Et_2O (3 × 30 mL). The combined organic layers were concentrated under reduced pressure, and the residue was purified by flash column chromatography (silica gel, EtOAc:hexane = 5:95) to give **S11** as a colourless oil (2.61 g, 46%). ***R*~F~** (EtOAc:hexane = 1:4) 0.34. **[α]$_D^{20}$** +2.0 (*c* 2.0, EtOAc) (lit.[2b] **[α]$_D^{21}$** +8.8 ± 0.1 (*c* 2.0, EtOAc)). **IR** ν_{max}/cm^{-1} 3497, 2959, 2873, 1734, 1468, 1448, 1370, 1300, 1218, 1182, 1144, 1096, 1029, 859. **^{1}H NMR** (300 MHz, $CDCl_3$) δ 0.93 (d, 3H, *J* 6.3, CH_3CHCH_3), 0.94 (d, 3H, *J* 6.0, CH_3CHCH_3), 1.24 (t, 3H, *J* 7.2, $COOCH_2CH_3$), 1.31 (t, 3H, *J* 7.2, $COOCH_2CH_3$), 1.46-1.55 (m, 1H, $CHHCH(CH_3)_2$), 1.60-1.81 (m, 2H, $CHHCH(CH_3)_2$), 2.93 (dt, 1H, *J* 6.9 & 3.6, $COCHCH_2$), 3.19 (d, 1H, *J* 7.8, OH), 4.14 (q, 2H, *J* 7.2, $COOCH_2CH_3$), 4.20-4.31 (m, 3H, $COOCH_2CH_3$ & COCHOH). **^{13}C NMR** (75 MHz, $CDCl_3$) δ 14.1, 22.3, 22.4, 25.7, 36.9, 46.6, 60.7, 61.7, 71.3, 173.0, 173.4 (one carbon signal overlapping or obscured). **MS** (ESI) *m/z* 268.7 ([M+Na]$^+$, 100%), 514.4 ([2M+Na]$^+$, 63%). The spectroscopic data were in agreement with those in the literature.[2b,5]

(2*S*,3*R*)-2-Hydroxy-3-isobutylsuccinic acid (S12).[2b]

To a solution of compound **S11** (143 mg, 0.580 mmol) in H_2O/1,4-dioxane (12 mL/8 mL) was added NaOH pellets (500 mg, 12.5 mmol). The reaction mixture was stirred at room temperature for 19 h and washed with Et_2O (2 × 5 mL). The aqueous solution was

taken to pH 1 with 6 M HCl and extracted with Et_2O (6 × 20 mL). The combined organic layers were dried over Na_2SO_4 and concentrated under reduced pressure and *in vacuo* to give **S12** as a yellow viscous oil (110 mg, 100%). **$[\alpha]_D^{20}$** +17.0 (*c* 0.5, EtOAc) (lit.[2b] **$[\alpha]_D^{21}$** +18.7 ± 0.2 (*c* 0.5, EtOAc)). **IR** ν_{max}/cm^{-1} 3439, 3396, 3348, 3193, 3102, 2958, 2876, 2605, 1714, 1457, 1379, 1202, 1146, 1094, 908, 811, 723, 640, 560. **^{1}H NMR** (300 MHz, $CDCl_3$) δ 0.94 (d, 3H, *J* 5.4, C*H*$_3$CHCH$_3$), 0.96 (d, 3H, *J* 5.4, CH$_3$CHC*H*$_3$), 1.45-1.90 (m, 3H, C*H*$_2$C*H*(CH$_3$)$_2$), 2.95-3.20 (m, 1H, COC*H*CH$_2$), 4.31 (d, 1H, *J* 3.0, COC*H*OH), 7.41 (br s, 2H, 2 × COOH) (one hydroxyl proton signal (OH) not observed). **^{13}C NMR** (75 MHz, $CDCl_3$) δ 22.2, 22.6, 25.7, 36.8, 46.3, 70.7, 177.7, 178.6. **MS** (ESI) *m/z* 188.9 ([M-H]$^-$, 100%). The spectroscopic data were in agreement with those in the literature.[2b]

(*R*)-2-((*S*)-2,2-Dimethyl-5-oxo-1,3-dioxolan-4-yl)-4-methylpentanoic acid (6).[6]

To a solution of compound **S12** (2.51 g, 13.2 mmol) in 2,2-dimethoxypropane (25 mL) was added TsOH (251 mg, 13.2 mmol). The reaction mixture was stirred at room temperature for 2.5 h and concentrated under reduced pressure. The residue was diluted with EtOAc (45 mL) and washed with H_2O (3 × 10 mL) and brine (10 mL). The organic layer was concentrated under reduced pressure, and the residue was purified by flash column chromatography (silica gel, DCM ramping to DCM:CH_3OH = 98.5:1.5) to give **6** as a dark yellow viscous oil (2.57 g, 85%). ***R*$_F$** (DCM:CH_3OH = 9:1) 0.52. **$[\alpha]_D^{20}$** +1.8 (*c* 1.0, $CHCl_3$). **IR** ν_{max}/cm^{-1} 3175, 2958, 2874, 2634, 1791, 1716, 1461, 1383, 1264, 1220, 1119, 984, 942, 891. **^{1}H NMR** (300 MHz, $CDCl_3$) δ 0.95 (d, 6H, *J* 4.8, CH(C*H*$_3$)$_2$), 1.55 (s, 3H, C*H*$_3$CCH$_3$), 1.60 (s, 3H, CH$_3$CC*H*$_3$), 1.63-1.84 (m, 3H, C*H*$_2$C*H*(CH$_3$)$_2$), 2.97-3.04 (m, 1H, COC*H*CH$_2$), 4.48 (d, 1H, *J* 4.5, COC*H*CHCH$_2$), 8.75 (br s, 1H, COOH). **^{13}C NMR** (75 MHz, $CDCl_3$) δ 22.1, 22.5, 25.7, 26.1, 26.5, 36.5, 45.0, 74.1, 111.2, 172.0, 177.0. **MS** (ESI) *m/z* 252.7 ([M+Na]$^+$, 100%). The spectroscopic data were in agreement with those in the literature.[6-7]

4. Synthesis of Marimastat Derivatives 9 and 12-14

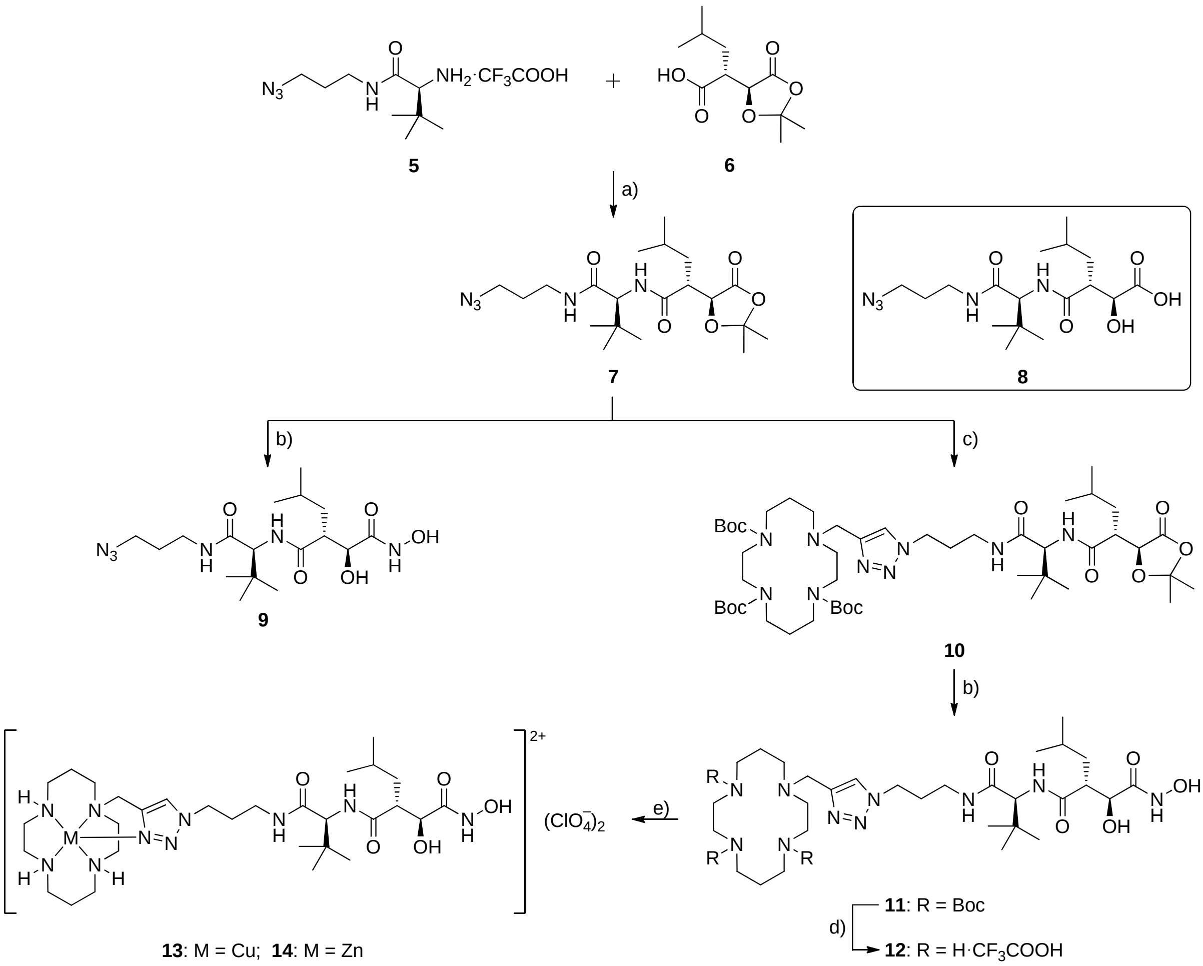

Scheme S3. Synthesis of the cyclam-marimastat conjugate (**12**) and its metal complexes (**13** and **14**) as well as azide-capped marimastat (**9**). Reagents and conditions: (a) EDC·HCl, HOBt, DIPEA, DCM, rt, 2.5 h, 73%; (b) 50% NH_2OH/H_2O, THF, reflux, 1 h, **9**: 81%, **11**: 83%; (c) propargyl-tri-Boc cyclam, $CuSO_4·H_2O$, sodium ascorbate, *t*BuOH/H_2O (1:1), rt, 1.5 h, 82%; (d) TFA/DCM/H_2O (90:5:5), rt, 6 h, followed by RP-HPLC purification, 95%; (e) $M(ClO_4)_2·6H_2O$ (M = Cu or Zn), EtOH, reflux, 3 h, **13**: 63%, **14**: 89%.

(*R*)-*N*-((*S*)-1-((3-Azidopropyl)amino)-3,3-dimethyl-1-oxobutan-2-yl)-2-((*S*)-2,2-dimethyl-5-oxo-1,3-dioxolan-4-yl)-4-methylpentanamide (7).

To a solution of compound **6** (136 mg, 0.591 mmol), EDC·HCl (124 mg, 0.647 mmol), HOBt (88.1 mg, 0.652 mmol) and DIPEA (310 µL, 1.78 mmol) in DCM (10 mL) was added a solution of compound **5** (193 mg, 0.590 mmol) and DIPEA (310 µL, 1.78 mmol) in DCM (10 mL). The reaction mixture was stirred at room temperature for 2 h and concentrated under reduced pressure. The residue was purified by flash column

chromatography (silica gel, DCM:EtOAc = 95:5 ramping to 92.5:7.5) to yield a pale yellow solid, which was recrystallized (EtOAc:hexane = 1:20) to give **7** as a white solid (182 mg, 73%). $\boldsymbol{R}_{\mathbf{F}}$ (DCM:EtOAc = 4:1) 0.54. **m.p.** 176-177 °C. $[\alpha]_D^{20}$ -6.2 (*c* 1.0, $CHCl_3$). **IR** ν_{max}/cm^{-1} 3319, 2956, 2874, 2095, 1784, 1641, 1531, 1464, 1375, 1259, 1125, 984, 895, 756, 666, 627, 550. **^{1}H NMR** (300 MHz, $CDCl_3$) δ 0.91 (d, 3H, *J* 5.7, CH_3CHCH_3), 0.93 (d, 3H, *J* 5.7, CH_3CHCH_3), 1.01 (s, 9H, $C(CH_3)_3$), 1.54 (s, 3H, CH_3CCH_3), 1.58-1.73 (m, 3H, $CH_2CH(CH_3)_2$), 1.64 (s, 3H, CH_3CCH_3), 1.75-1.85 (m, 2H, $CH_2CH_2CH_2$), 2.78-2.86 (m, 1H, $CHCH_2CH(CH_3)_2$), 3.19-3.43 (m, 2H, $CONHCH_2$), 3.34 (t, 2H, *J* 6.6, CH_2N_3), 4.38 (d, 1H, *J* 9.3, $CHC(CH_3)_3$), 4.48 (d, 1H, *J* 5.7, COC*H*CHCONH), 6.81 (d, 1H, *J* 9.3, CHCON*H*CH), 7.18 (t, 1H, *J* 5.7, CHCON*H*CH_2). **^{13}C NMR** (75 MHz, $CDCl_3$) δ 22.0, 23.1, 25.7, 25.8, 26.7, 28.9, 34.6, 36.8, 37.2, 47.2, 49.2, 60.6, 74.7, 111.0, 170.6, 170.8, 171.9 (three carbon signals overlapping or obscured). **MS** (ESI) *m/z* 447.9 ($[M+Na]^+$, 100%), 872.7 ($[2M+Na]^+$, 13%). **HRMS** (ESI) 448.25340 ($[M+Na]^+$); calcd. for $C_{20}H_{35}N_5NaO_5$ ($[M+Na]^+$) 448.25304.

(2*S*,3*R*)-3-(((*S*)-1-((3-Azidopropyl)amino)-3,3-dimethyl-1-oxobutan-2-yl)carbamoyl)-2-hydroxy-5-methylhexanoic acid (8).

Compound **8** was obtained as a white solid (13 mg, 5%) in the above synthetic procedure of **7**. $\boldsymbol{R}_{\mathbf{F}}$ (DCM:CH_3OH = 9:1) 0.12. **m.p.** 166-168 °C. $[\alpha]_D^{20}$ -28.0 (*c* 1.0, CH_3OH). **IR** ν_{max}/cm^{-1} 3305, 2956, 2875, 2473, 2425, 2096, 1707, 1621, 1445, 1369, 1230, 1094. **^{1}H NMR** (400 MHz, CD_3OD) δ 0.90 (d, 3H, *J* 5.2, CH_3CHCH_3), 0.92 (d, 3H, *J* 5.2, CH_3CHCH_3), 1.01 (s, 9H, $C(CH_3)_3$), 1.23-1.37 (m, 1H, C*H*HCH$(CH_3)_2$), 1.45-1.60 (m, 1H, $CH_2CH(CH_3)_2$), 1.66-1.82 (m, 3H, CH*H*CH$(CH_3)_2$ & $CH_2CH_2CH_2$), 2.82-2.92 (m, 1H, $CHCH_2CH(CH_3)_2$), 3.26 (t, 2H, *J* 6.8, $CH_2CH_2CH_2$), 3.34 (t, 2H, *J* 6.8, $CH_2CH_2CH_2$), 4.23 (s, 1H, $CHC(CH_3)_3$), 4.29 (d, 1H, *J* 5.2, C*H*OH) (two amide proton signals (2 × CONH), one carboxylic acid proton signal (COOH) and one hydroxyl proton signal (OH) not observed due to H/D exchange). **^{13}C NMR** (100 MHz, CD_3OD) δ 21.9, 24.0, 26.9, 27.3, 29.5, 35.0, 37.5, 37.8, 49.1, 62.2, 72.5, 172.6, 175.4, 175.9 (three carbon signals overlapping or obscured). **MS** (ESI) *m/z* 385.9 ($[M+H]^+$, 17%), 407.9 ($[M+Na]^+$, 37%), 792.9 ($[2M+Na]^+$, 100%). **HRMS** (ESI) 408.22182 ($[M+Na]^+$); calcd. for $C_{17}H_{31}N_5NaO_5$ ($[M+Na]^+$) 408.22174.

(2*R*,3*S*)-*N*¹-((*S*)-1-((3-Azidopropyl)amino)-3,3-dimethyl-1-oxobutan-2-yl)-*N*⁴,3-dihydroxy-2-isobutylsuccinamide (9). [8]

To a solution of acetonide **7** (213 mg, 0.501 mmol) in THF (10 mL) was added hydroxylamine (50% in H_2O, 1.53 mL, 25.0 mmol). The reaction was heated at reflux for 1 h and concentrated under reduced pressure. The residue was purified by preparative RP-HPLC purification (gradient 0% to 40% B over 45 min) to give **9** as a white solid (163 mg, 81%). $\boldsymbol{R_F}$ (DCM:CH_3OH = 9:1) 0.16. **m.p.** 71-72 °C. $[\alpha]_D^{20}$ -24.1 (*c* 1.0, CH_3OH). **IR** ν_{max}/cm^{-1} 3292, 3219, 2959, 2874, 2098, 1634, 1531, 1466, 1439, 1368, 1246, 1194, 1138, 1082, 836, 803, 721, 643. **^{1}H NMR** (400 MHz, DCON($CD_3)_2$) δ 0.83 (d, 3H, *J* 6.8, C*H*$_3$CHCH$_3$), 0.87 (d, 3H, *J* 6.8, CH$_3$CHC*H*$_3$), 0.99 (s, 9H, C(CH$_3$)$_3$), 1.12-1.24 (m, 1H, C*H*HCH(CH$_3$)$_2$), 1.45-1.58 (m, 1H, CH$_2$C*H*(CH$_3$)$_2$), 1.58-1.68 (m, 1H, CH*H*CH(CH$_3$)$_2$), 1.71-1.83 (m, 2H, CH$_2$C*H*$_2$CH$_2$), 2.84-2.93 (m, 1H, C*H*CH$_2$CH(CH$_3$)$_2$), 3.21-3.36 (m, 2H, CONHC*H*$_2$), 3.42 (t, 2H, *J* 7.2, CH$_2$N$_3$), 4.04 (apparent t, 1H, *J* 6.8, COC*H*OH), 4.28 (d, 1H, *J* 9.6, C*H*C(CH$_3$)$_3$), 5.73 (d, 1H, *J* 7.6, COCHO*H*), 7.78 (d, 1H, *J* 9.2, CHCON*H*CH), 8.06 (t, 1H, *J* 5.6, CON*H*CH$_2$), 9.21 (s, 1H, CONHO*H*), 10.72 (s, 1H, CON*H*OH). **^{13}C NMR** (75 MHz, $(CD_3)_2SO$) δ 21.8, 23.5, 25.4, 26.7, 28.4, 34.1, 35.6, 37.4, 47.9, 48.3, 60.1, 71.4, 168.9, 170.2, 172.4 (two carbon signals overlapping or obscured). **MS** (ESI) *m/z* 399.1 ([M-H]$^-$, 100%). **HRMS** (ESI) 423.23237 ([M+Na]$^+$); calcd. for $C_{17}H_{32}N_6NaO_5$ ([M+Na]$^+$) 423.23264.

Tri-*tert*-butyl 11-((1-(3-((*S*)-2-((*R*)-2-((*S*)-2,2-dimethyl-5-oxo-1,3-dioxolan-4-yl)-4-methylpentanamido)-3,3-dimethylbutanamido)propyl)-1*H*-1,2,3-triazol-4-yl)methyl)-1,4,8,11-tetraazacyclotetradecane-1,4,8-tricarboxylate (10).

Propargyl-tri-Boc cyclam[9] (108 mg, 0.200 mmol) and azide **7** (85.1 mg, 0.200 mmol) were dissolved in *t*BuOH/H_2O (10 mL: 8 mL). A brown cloudy solution of $CuSO_4\cdot5H_2O$ (2.5 mg, 0.010 mmol, 5 mol%) and sodium ascorbate (4.0 mg, 0.020 mmol, 10 mol%) in H_2O (2 mL) was added. The reaction mixture was stirred at room temperature for 1.5 h, quenched with 5% $NaHCO_3$ (5 mL) and extracted with EtOAc (3 × 25 mL). The combined organic layers were concentrated under reduced pressure, and the residue was purified by flash column chromatography (silica gel, EtOAc:hexane = 4:1 ramping to EtOAc) to give **10** as a white foam (158 mg, 82%). $\boldsymbol{R_F}$ (EtOAc) 0.68. $[\alpha]_D^{20}$ -1.4 (*c* 1.0, $CHCl_3$). **IR** ν_{max}/cm^{-1} 3329, 2966, 2876, 1789, 1686, 1533, 1467, 1413, 1370, 1248, 1163,

1051, 985, 898, 774, 733. **^{1}H NMR** (300 MHz, $CDCl_3$) δ 0.90 (d, 3H, *J* 5.4, CH_3CHCH_3), 0.92 (d, 3H, *J* 5.1, CH_3CHCH_3), 1.02 (s, 9H, $CHC(CH_3)_3$), 1.43 (s, 9H, $COOC(CH_3)_3$), 1.46 (s, 18H, 2 × $COOC(CH_3)_3$), 1.55 (s, 3H, CH_3CCH_3), 1.58-1.78 (m, 5H, $CH_2CH(CH_3)_2$ & $CH_2NCH_2CH_2CH_2NCH_2$), 1.64 (s, 3H, CH_3CCH_3), 1.80-2.00 (m, 2H, $CH_2NCH_2CH_2CH_2NCH_2$), 2.05-2.20 (m, 2H, triazole-$CH_2CH_2CH_2NHCO$), 2.38-2.52 (m, 2H, CH_2N(CH_2-triazole)CH_2), 2.58-2.68 (m, 2H, CH_2N(CH_2-triazole)CH_2), 2.75-2.85 (m, 1H, $CHCH_2CH(CH_3)_2$), 3.18-3.34 (br m, 14H, 3 × $CH_2N(Boc)CH_2$ & CH_2NHCO), 3.76 (br s, 2H, NCH_2-triazole), 4.21 (d, 1H, *J* 8.7, $CHC(CH_3)_3$), 4.37 (t, 2H, *J* 6.6, triazole-CH_2CH_2), 4.54 (d, 1H, *J* 5.4, NHCOCHCHCO), 6.69 (br d, 2H, *J* 8.7, NHCOCHNHCO), 7.54 (br s, 1H, triazole-H). **^{13}C NMR** (75 MHz, $CDCl_3$) δ 22.0, 23.2, 25.8, 26.0, 26.8, 28.6, 30.1, 34.4, 36.4, 37.1, 45.5, 47.3, 47.5, 48.8, 51.1, 61.2, 74.8, 79.7, 111.1, 123.1, 143.8, 155.6, 155.9, 170.8, 170.9, 172.2 (twenty two carbon signals overlapping or obscured). **MS** (ESI) *m/z* 964.3 ($[M+H]^+$, 22%), 986.4 ($[M+Na]^+$, 100%). **HRMS** (ESI) 986.62601 ($[M+Na]^+$); calcd. for $C_{48}H_{85}N_9NaO_{11}$ ($[M+Na]^+$) 986.62608.

Tri-*tert*-butyl 11-((1-(3-((*S*)-2-((*R*)-2-((*S*)-1-hydroxy-2-(hydroxyamino)-2-oxoethyl)-4-methylpentanamido)-3,3-dimethylbutanamido)propyl)-1*H*-1,2,3-triazol-4-yl)methyl)-1,4,8,11-tetraazacyclotetradecane-1,4,8-tricarboxylate (11).[8]

To a solution of acetonide **10** (804 mg, 0.834 mmol) in THF (40 mL) was added hydroxylamine (50% in H_2O, 2.56 mL, 41.8 mmol). The reaction was heated at reflux for 1 h and concentrated under reduced pressure. The residue was purified by flash column chromatography (silica gel, DCM:CH_3OH = 98:2 ramping to 90:10) to give **11** as a white foam (648 mg, 83%). ***R*$_F$** (DCM:CH_3OH = 9:1) 0.41. **$[\alpha]_D^{20}$** -24.6 (*c* 1.0, $CHCl_3$). **IR** ν_{max}/cm^{-1} 3275, 3074, 2966, 1673, 1643, 1543, 1468, 1415, 1368, 1310, 1244, 1159, 914, 730. **^{1}H NMR** (300 MHz, $CDCl_3$) δ 0.91 (d, 3H, *J* 5.4, CH_3CHCH_3), 0.93 (d, 3H, *J* 5.4, CH_3CHCH_3), 1.00 (s, 9H, $CHC(CH_3)_3$), 1.46 (s, 27H, 3 × $COOC(CH_3)_3$), 1.51-1.80 (m, 5H, $CH_2CH(CH_3)_2$ & $CH_2NCH_2CH_2CH_2NCH_2$), 1.80-2.00 (m, 2H, $CH_2NCH_2CH_2CH_2NCH_2$), 2.02-2.19 (m, 2H, triazole-$CH_2CH_2CH_2NHCO$), 2.35-2.55 (m, 2H, CH_2N(CH_2-triazole)CH_2), 2.55-2.75 (m, 2H, CH_2N(CH_2-triazole)CH_2), 3.04-3.17 (m, 1H, $CHCH_2CH(CH_3)_2$), 3.20-3.55 (br m, 14H, 3 × $CH_2N(Boc)CH_2$ & CH_2NHCO), 3.82 (br s, 2H, NCH_2-triazole), 4.16 (d, 1H, *J* 8.7, $CHC(CH_3)_3$), 4.30 (s, 1H, CHCONHOH), 4.34-4.49 (m, 2H, triazole-CH_2CH_2), 5.72 (br s, 1H, CHOH), 7.36 (br s, 1H, CH_2NHCO), 7.77 (br d, 1H, *J* 8.1, CHNHCOCH), 7.89 (br s, 1H,

triazole-H), 9.27 (br s, 1H, CONHO*H*), 9.81 (br s, 1H, CON*H*OH). **^{13}C NMR** (75 MHz, $CDCl_3$) δ 22.4, 22.9, 25.8, 27.0, 28.6, 30.2, 34.5, 36.4, 39.1, 45.5, 47.3, 47.6, 50.7, 52.0, 61.4, 71.4, 79.8, 123.8, 142.7, 155.8, 156.0, 169.7, 171.6, 174.5 (twenty one carbon signals overlapping or obscured). **MS** (ESI) *m/z* 939.4 ([M+H]$^+$, 100%), 961.4 ([M+Na]$^+$, 32%). **HRMS** (ESI) 939.62451 ([M+H]$^+$); calcd. for $C_{45}H_{83}N_{10}O_{11}$ ([M+H]$^+$) 939.62373.

11-((1-(3-((*S*)-2-((*R*)-2-((*S*)-1-Hydroxy-2-(hydroxyamino)-2-oxoethyl)-4-methylpentanamido)-3,3-dimethylbutanamido)propyl)-1*H*-1,2,3-triazol-4-yl)methyl)-11-aza-1,4,8-triazoniacyclotetradecane-1,4,8-triium 2,2,2-trifluoroacetate (12).

Compound **11** (188 mg, 0.200 mmol) was dissolved in a mixture of TFA/DCM/H_2O (90:5:5, 20 mL). The reaction mixture was stirred at room temperature for 6 h and concentrated under reduced pressure. The residue was purified by preparative RP-HPLC purification (5% B over 15 min, followed by gradient 5% to 25% B over 45 min) to give **12** as a white solid (186 mg, 95%). **m.p.** 50-51 °C. **$[\alpha]_D^{20}$** -7.8 (*c* 1.0, H_2O). **IR** ν_{max}/cm^{-1} 3279, 3090, 2965, 2860, 1666, 1538, 1434, 1377, 1188, 1132, 800, 719. **^{1}H NMR** (300 MHz, D_2O) δ 0.85 (d, 3H, *J* 6.6, C*H*$_3$CHCH$_3$), 0.90 (d, 3H, *J* 6.6, CH$_3$CHC*H*$_3$), 1.03 (s, 9H, C(CH$_3$)$_3$), 1.16-1.25 (m, 1H, C*H*HCH(CH$_3$)$_2$), 1.37-1.50 (m, 1H, CH$_2$C*H*(CH$_3$)$_2$), 1.57-1.67 (m, 1H, CH*H*CH(CH$_3$)$_2$), 1.82-1.97 (m, 2H, CH$_2$NCH$_2$C*H*$_2$CH$_2$NCH$_2$), 2.02-2.23 (m, 4H, triazole-CH$_2$C*H*$_2$CH$_2$NHCO & CH$_2$NCH$_2$C*H*$_2$CH$_2$NCH$_2$), 2.80 (t, 2H, *J* 5.1, C*H*$_2$N(CH$_2$-triazole)CH$_2$), 2.86 (t, 2H, *J* 5.1, CH$_2$N(CH$_2$-triazole)C*H*$_2$), 2.91-3.10 (m, 5H), 3.10-3.21 (m, 4H), 3.21-3.40 (m, 6H) (total 15H, C*H*CH$_2$CH(CH$_3$)$_2$ & 3 × C*H*$_2$NH$_2^+$C*H*$_2$ & C*H*$_2$NHCO), 3.84 (br s, 2H, NC*H*$_2$-triazole), 4.16 (d, 1H, *J* 7.8, CHC*H*CONHOH), 4.17 (s, 1H, C*H*C(CH$_3$)$_3$), 4.47 (t, 2H, *J* 6.9, triazole-C*H*$_2$CH$_2$), 7.97 (s, 1H, triazole-H) (six ammonium proton signals (3 × NH$_2^+$), two amide proton signals (2 × CONH), one hydroxyl proton signal (OH) and two hydroxamic acid proton signals (CONHOH) not observed due to H/D exchange). **^{13}C NMR** (75 MHz, D_2O) δ 22.0, 22.4, 22.6, 23.4, 26.3, 26.8, 29.9, 34.2, 36.7, 38.2, 41.6, 43.7, 45.6, 47.2, 48.5, 48.7, 50.2, 52.3, 62.9, 72.3, 117.2 (q, $J_{C\text{-}F}$ 292.5, 3 × CF$_3$), 126.2, 142.9, 163.7 (q, $J_{C\text{-}F}$ 37.5, 3 × CF$_3$*C*OOH), 171.2, 173.1, 176.1 (five carbon signals overlapping or obscured). **MS** (ESI) *m/z* 639.5 ([M-3TFA+H]$^+$, 100%), 661.5 ([M-3TFA+Na]$^+$, 20%). **HRMS** (ESI) 639.46530 ([M-3TFA+H]$^+$); calcd. for $C_{30}H_{59}N_{10}O_5$ ([M-3TFA+H]$^+$) 639.46644. **Anal.** Calcd. for $C_{36}H_{61}F_9N_{10}O_{11}$: C 44.08, H 6.27, N 14.28; Found: C 43.85, H 6.35, N 14.40.

CAUTION! Perchlorate salts of metal complexes with organic ligands are potentially explosive and should be handled with care. Only small amounts of material should be prepared.

[Cu(12-3TFA)](ClO_4)$_2$ complex (13).

To a solution of compound **12** (113 mg, 0.115 mmol) in EtOH (1 mL) was added dropwise a solution of $Cu(ClO_4)_2{\cdot}6H_2O$ (42.7 mg, 0.115 mmol) in EtOH (2 mL) at room temperature. The reaction mixture was heated at reflux for 3 h, cooled on an ice bath and the solvent was decanted. The remaining residue was washed with ice-cold EtOH (3 × 1 mL) and Et_2O (3 × 3 mL), and dried *in vacuo* to give **13** as a purple powder (65.1 mg, 63%). **m.p.** 140-145 °C. **$[\alpha]_D^{20}$** -10.0 (*c* 0.1, H_2O). **UV-Vis** (CH_3OH) λ_{max}/nm 588, ε 150. **IR** ν_{max}/cm^{-1} 3565, 3377, 3240, 2957, 2881, 1647, 1533, 1468, 1368, 1234, 1199, 1087, 625. **HRMS** (ESI) 800.33675, 801.34057, 802.33528, 803.33839, 804.33407, 805.33680 ([M-ClO_4]$^+$); calcd. for $C_{30}H_{58}ClCuN_{10}O_9$ ([M-ClO_4]$^+$) 800.33673, 801.34001, 802.33500, 803.33820, 804.33214, 805.33534. **Anal.** Calcd. for $C_{30}H_{58}Cl_2CuN_{10}O_{13}{\cdot}2H_2O$: C 38.44, H 6.67, N 14.94; Found: C 38.49, H 6.60, N 14.85.

[Zn(12-3TFA)](ClO_4)$_2$ complex (14).

To a solution of compound **12** (114 mg, 0.116 mmol) in EtOH (1 mL) was added dropwise a solution of $Zn(ClO_4)_2{\cdot}6H_2O$ (43.3 mg, 0.116 mmol) in EtOH (2 mL) at room temperature. The reaction mixture was heated at reflux for 3 h, cooled on an ice bath and the solvent was decanted. The remaining residue was washed with ice-cold EtOH (3 × 1 mL) and Et_2O (3 × 3 mL), and dried *in vacuo* to give **14** as a white powder (93.2 mg, 89%). **m.p.** 70-75 °C. **$[\alpha]_D^{20}$** -6.0 (*c* 0.2, H_2O). **IR** ν_{max}/cm^{-1} 3575, 3503, 3371, 3249, 2956, 2877, 1645, 1533, 1462, 1367, 1232, 1198, 1079, 624. **HRMS** (ESI) 801.33647, 802.33888, 803.33451, 804.33607, 805.33198, 806.33511, 807.33177 ([M-ClO_4]$^+$); calcd. for $C_{30}H_{58}ClN_{10}O_9Zn$ ([M-ClO_4]$^+$) 801.33627, 802.33958, 803.33334, 804.33646, 805.33205, 806.33527, 807.32914. **Anal.** Calcd. for $C_{30}H_{58}Cl_2N_{10}O_{13}Zn{\cdot}3H_2O$: C 37.64, H 6.74, N 14.63; Found: C 37.29, H 6.41, N 14.47.

5. ^{1}H NMR Analysis for Recrystallization of Azide-Capped Marimastat 7

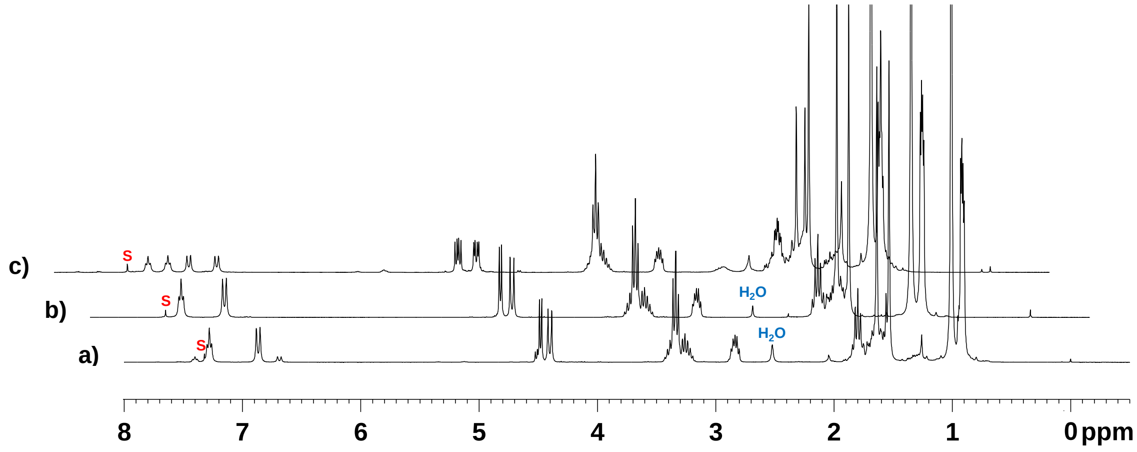

Figure S1. ^{1}H NMR spectra of **7** after purification by flash column chromatography (a) and recrystallization (b) compared to the ^{1}H NMR spectrum of the mother liquor concentrate (c) in $CDCl_3$. S: nondeuterated solvent residual peaks.

6. RP-HPLC Purification of the Cyclam-Marimastat Conjugate 12

Removal of the Boc groups from **11** was carried out in a mixture of TFA/DCM/H_2O (90:5:5). The crude products were initially purified by RP-HPLC using gradient 5% to 25% CH_3CN over 45 minutes, which gave excellent separation of a truncated cyclam-marimastat conjugate with loss of the succinate-derived moiety (**S13**, Figure S2(a)) from a mixture of the desired trifluoroacetate **12** and unidentified impurities. The complete separation of this mixture was ultimately achieved by eluting the column with 5% CH_3CN for 15 minutes prior to applying the aforementioned gradient (Figure S2(b)).

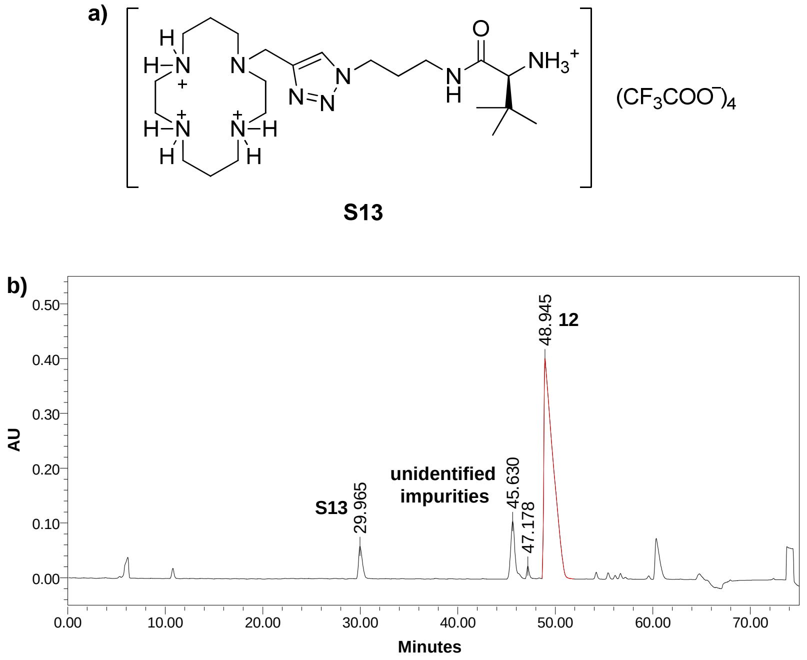

Figure S2. (a) Structure of byproduct **S13**; (b) Preparative RP-HPLC chromatogram for the separation of **12** (red) from **S13** and other impurities.

7. Distinct Colours Observed for Metal Complexation Titrations

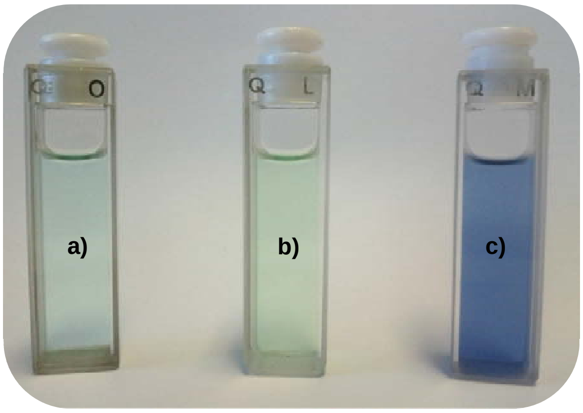

Figure S3. Distinct colours observed for metal complexation titrations: an equal amount of $Cu(ClO_4)_2$ (1.0 equivalent) in (a) CH_3OH – light blue; (b) a methanolic solution of **9** (5 mM) – pale green; (c) a methanolic solution of **12** (5 mM) – dark blue.

8. Cell Viability Assay

1 × 10^5 THP1 cells were seeded into 96 well plates and allowed to adhere. Compounds were serially diluted 1 in 10 with a starting concentration of 50 μM in 0.1% DMSO and added to the cells. Seven days post-treatment, cell viability was assessed by adding 0.05% resazurin to each well and measuring fluorescence using the FluoSTAR Omega microplate reader (λ_{ex} = 544 nm & λ_{em} = 595 nm). Percentage viability was calculated relative to a non-treated control and listed in Table S1.

Table S1. Cytotoxicity of the marimastat derivatives **7–9** and **11–14** against THP1.

**Compound**	**Drug Concentration**		
	**50 μM**	**5 μM**	**0.5 μM**
**7**	99.66±0.76	102.36±0.63	101.85±0.68
**8**	94.92±1.56	99.06±0.47	97.8±1.59
**9**	99.59±0.68	99.06±0.47	97.8±1.59
**11**	1.45±1.02	102.04±2.27	100.34±1.37
**12**	108.23±2.08	104.96±0.57	101.3±1.76
**13**	97.54±1.69	98.97±0.85	99.14±0.32
**14**	101.8±1.69	99.75±0.85	100.29±0.32

9. MMP Inhibition Assay

Catalytic domains of MMP-1 (MMP-1_{cat}), MMP-3 (MMP-3_{cat}) were prepared as previously described.[10] Fluorogenic MMP substrate Mca-Pro-Leu-Gly~Leu-Dpa-Ala-Arg-NH_2 (~ indicates the scissile bond, Mca = (7-methoxycoumarin-4-yl)-acetyl; Dpa = *N*-3-(2,4-dinitrophenyl)-L-α,β-diaminopropionyl)[11] was from Bachem, Bubendorf, Switzerland. Compounds **1**, **2**, **7–9** and **11–16** were pre-incubated with MMP-1 or MMP-3 (10 nM) at different concentrations (0–10 μM) in a mixture of Tris-HCl (50 mM, pH 7.5), NaCl (150 mM), $CaCl_2$ (10 mM), NaN_3 (0.02%) and Brij-35 (0.05%) for 1 hour at 37 °C. Residual activity was measured using the fluorogenic MMP substrate (2 μM) by fluorescence increase (emission at 393 nm and excitation at 325 nm) on a fluorescence plate reader (Spectramax, Molecular Devices, California, USA). The data were fitted to the tight binding inhibitor equation: $v = [(E\text{-}I\text{-}k+[(E\text{-}I\text{-}k)^2+4Ek]^{1/2})/(2E)]$, where v is the velocity of the reaction, E is the enzyme concentration, I is the initial inhibitor concentration, and k is the apparent inhibition constant, using the software Prism (Graphpad, La Jolla, USA).

10. References

[1] B. Yameen, M. Ali, M. Alvarez, R. Neumann, W. Ensinger, W. Knoll, O. Azzaroni, *Polym. Chem.* **2010**, *1*, 183-192.

[2] a) A. J. Vernall, S. Ballet, A. D. Abell, *Tetrahedron* **2008**, *64*, 3980-3997; b) K. Jenssen, K. Sewald, N. Sewald, *Bioconjugate Chem.* **2004**, *15*, 594-600.

[3] X. Zhou, W. J. Liu, J. L. Ye, P. Q. Huang, *Tetrahedron* **2007**, *63*, 6346-6357.

[4] a) K. Mori, H. Iwasawa, *Tetrahedron* **1980**, *36*, 87-90; b) T. H. T. Dang, L. de la Riva, R. P. Fagan, E. M. Storck, W. P. Heal, C. Janoir, N. F. Fairweather, E. W. Tate, *ACS Chem. Biol.* **2010**, *5*, 279-285.

[5] M. C. Pirrung, H. Han, D. S. Nunn, *J. Org. Chem.* **1994**, *59*, 2423-2429.

[6] D. E. Levy, F. Lapierre, W. Liang, W. Ye, C. W. Lange, X. Li, D. Grobelny, M. Casabonne, D. Tyrrell, K. Holme, A. Nadzan, R. E. Galardy, *J. Med. Chem.* **1998**, *41*, 199-223.

[7] W. Qiu, J. Xu, X. Li, L. Zhong, J. Li, J. Li, F. Nan, *Chin. J. Chem.* **2009**, *27*, 825-833.

[8] R. J. Davenport, R. J. Watson, *Tetrahedron Lett.* **2000**, *41*, 7983-7986.

[9] a) E. Tamanini, S. E. J. Rigby, M. Motevalli, M. H. Todd, M. Watkinson, *Chem. Eur. J.* **2009**, *15*, 3720-3728; b) M. Yu, J. R. Price, P. Jensen, C. J. Lovitt, T. Shelper, S. Duffy, L. C. Windus, V. M. Avery, P. J. Rutledge, M. H. Todd, *Inorg. Chem.* **2011**, *50*, 12823-12835; c) E. Tamanini, A. Katewa, L. M. Sedger, M. H. Todd, M. Watkinson, *Inorg. Chem.* **2009**, *48*, 319-324.

[10] a) L. Troeberg, M. Tanaka, R. Wait, Y. E. Shi, K. Brew, H. Nagase, *Biochemistry* **2002**, *41*, 15025-15035; b) Z. Yu, R. Visse, M. Inouye, H. Nagase, B. Brodsky, *J. Biol. Chem.* **2012**, *287*, 22988-22997.

[11] C. G. Knight, F. Willenbrock, G. Murphy, *FEBS Lett.* **1992**, *296*, 263-266.

11. ^{1}H & ^{13}C NMR Spectra for Novel Compounds

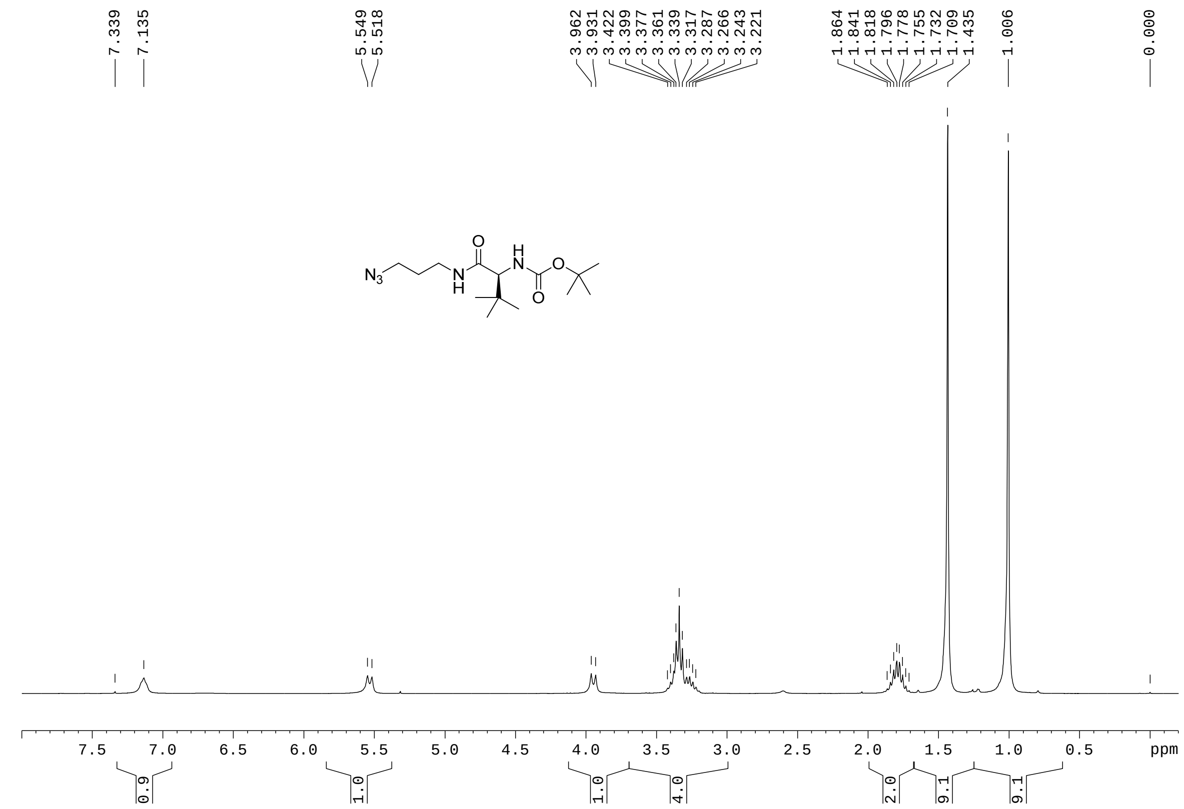

Figure S4. ^{1}H NMR spectrum (300 MHz) of **S5** in $CDCl_3$.

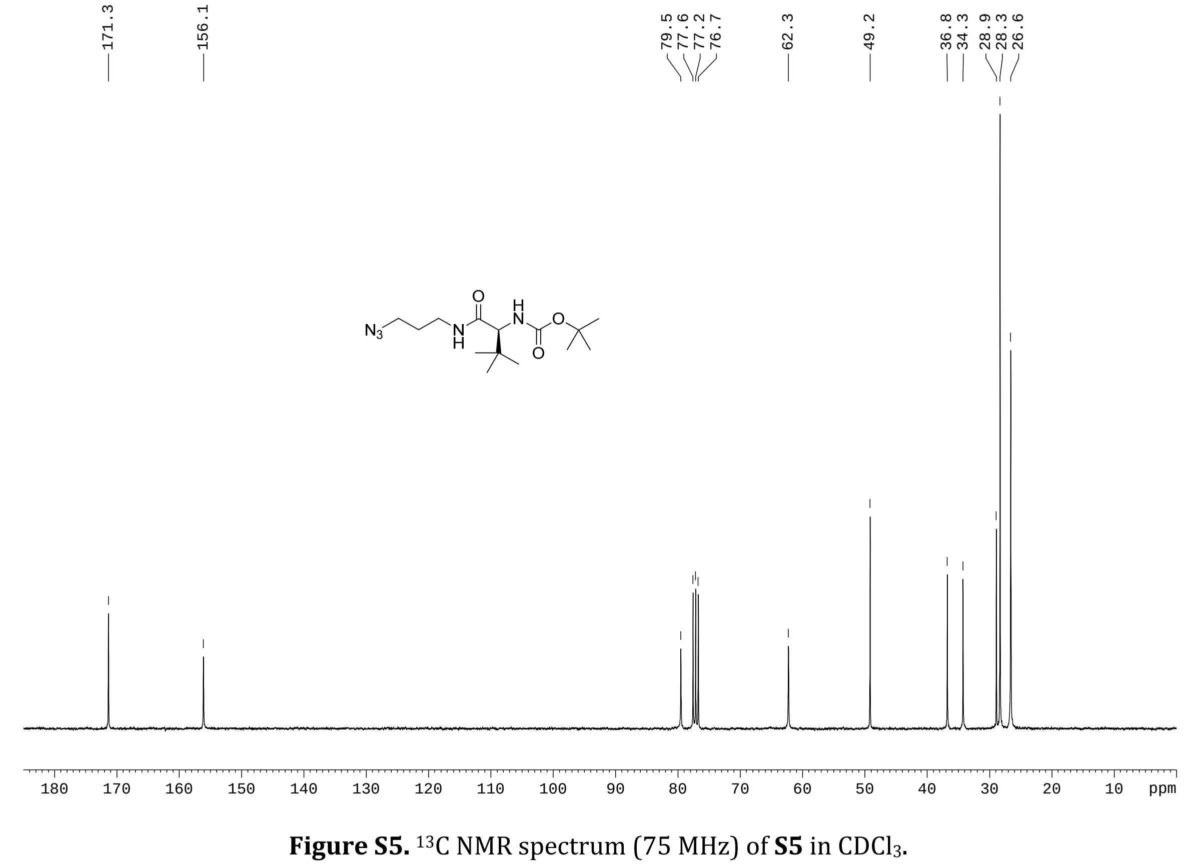

Figure S5. ^{13}C NMR spectrum (75 MHz) of **S5** in $CDCl_3$.

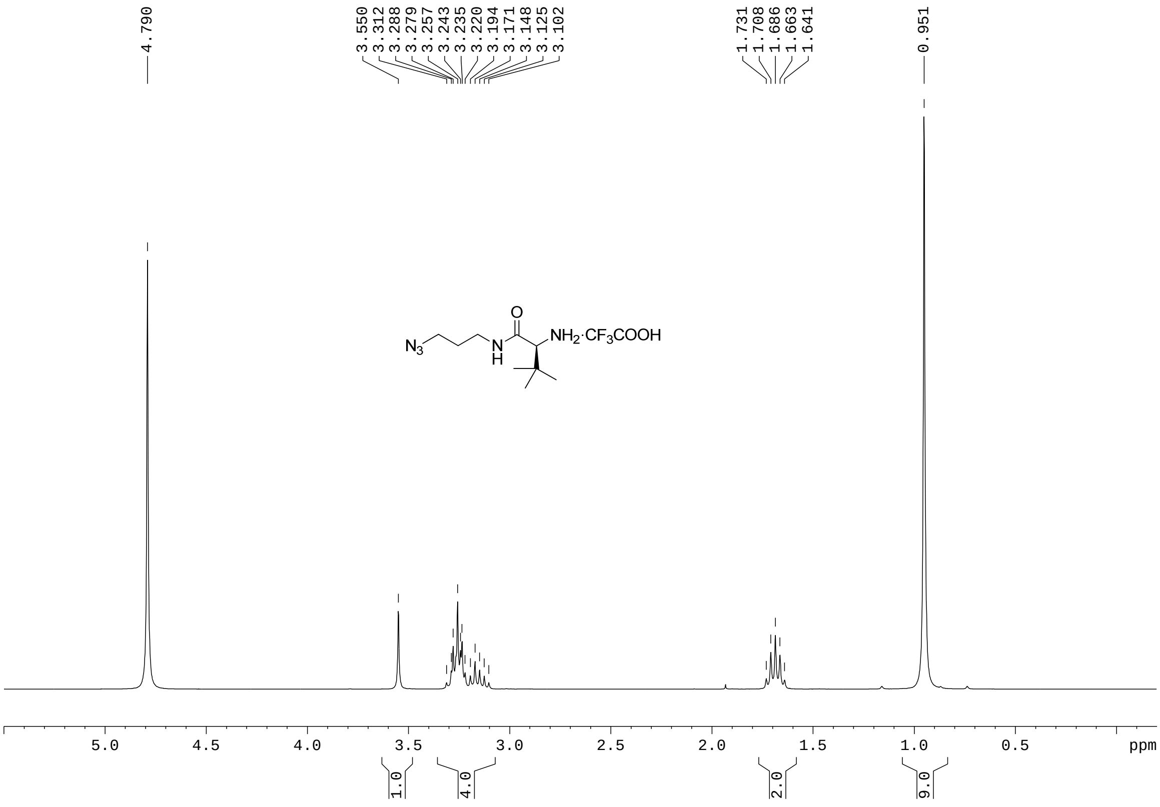

Figure S6. ^{1}H NMR spectrum (300 MHz) of **5** in D_2O.

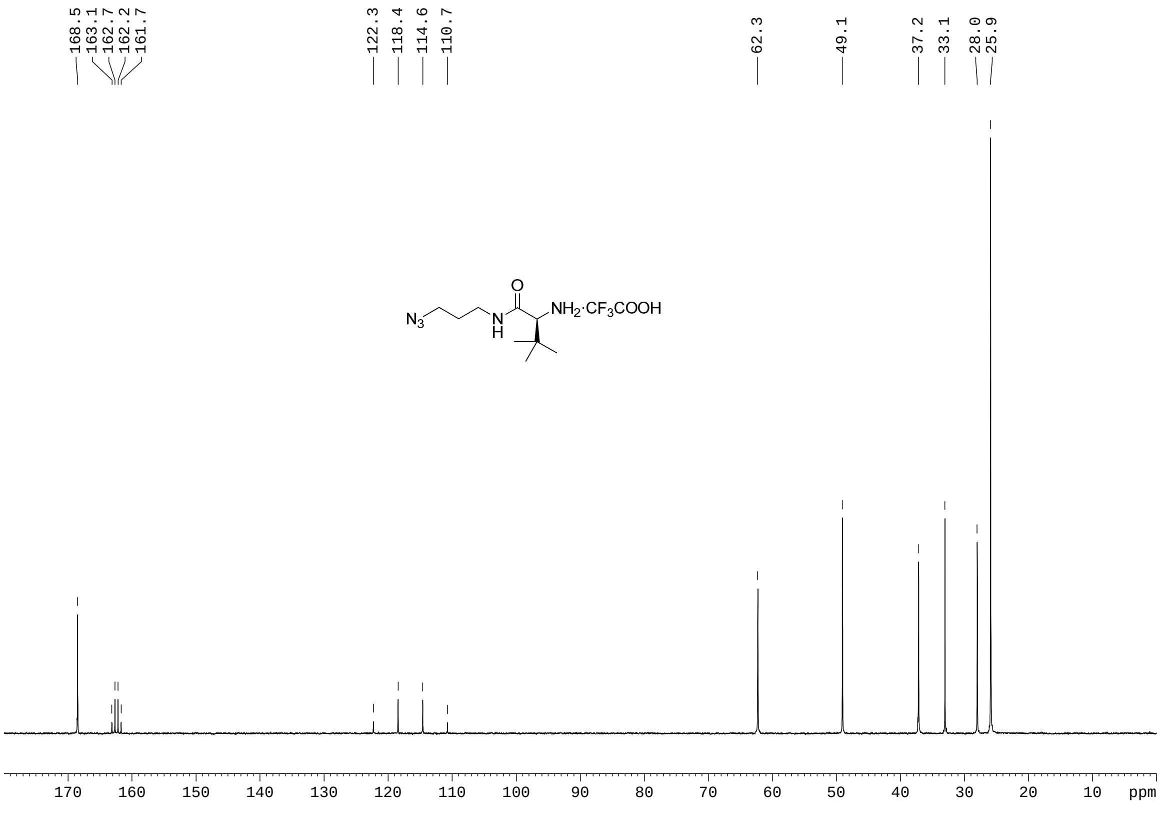

Figure S7. ^{13}C NMR spectrum (75 MHz) of **5** in D_2O.

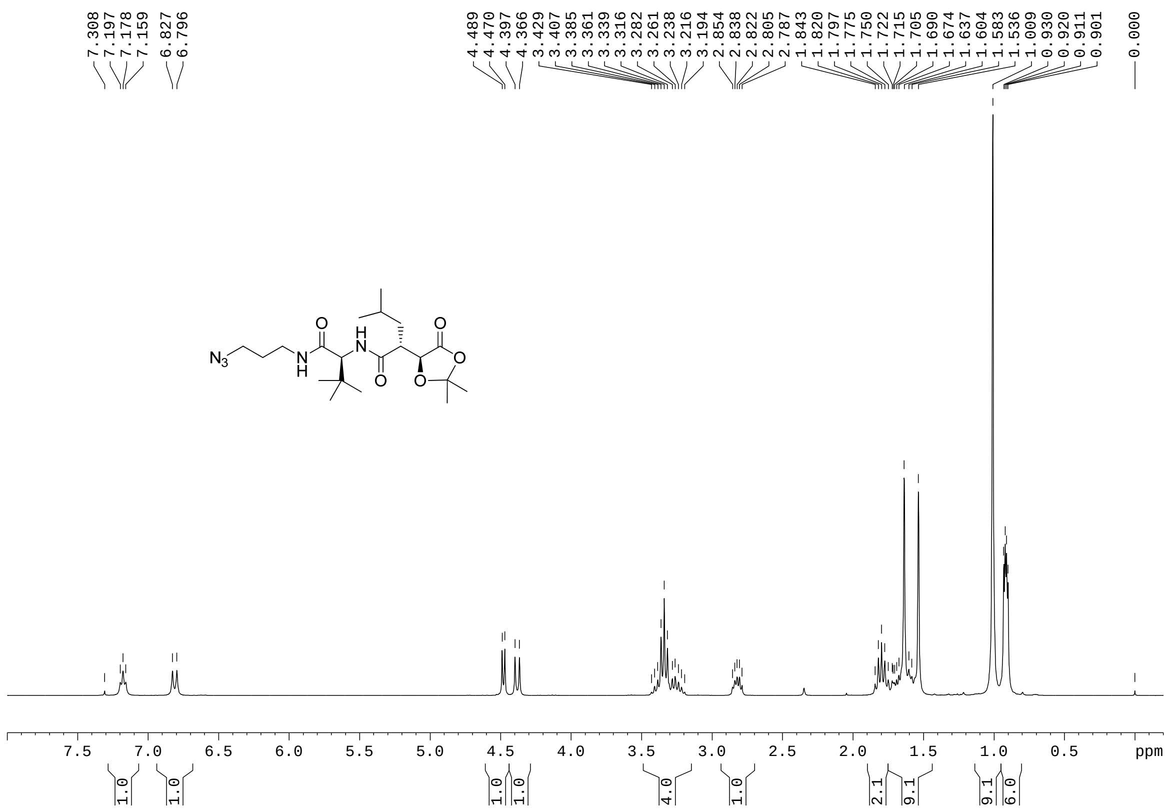

Figure S8. ^{1}H NMR spectrum (300 MHz) of **7** in $CDCl_3$.

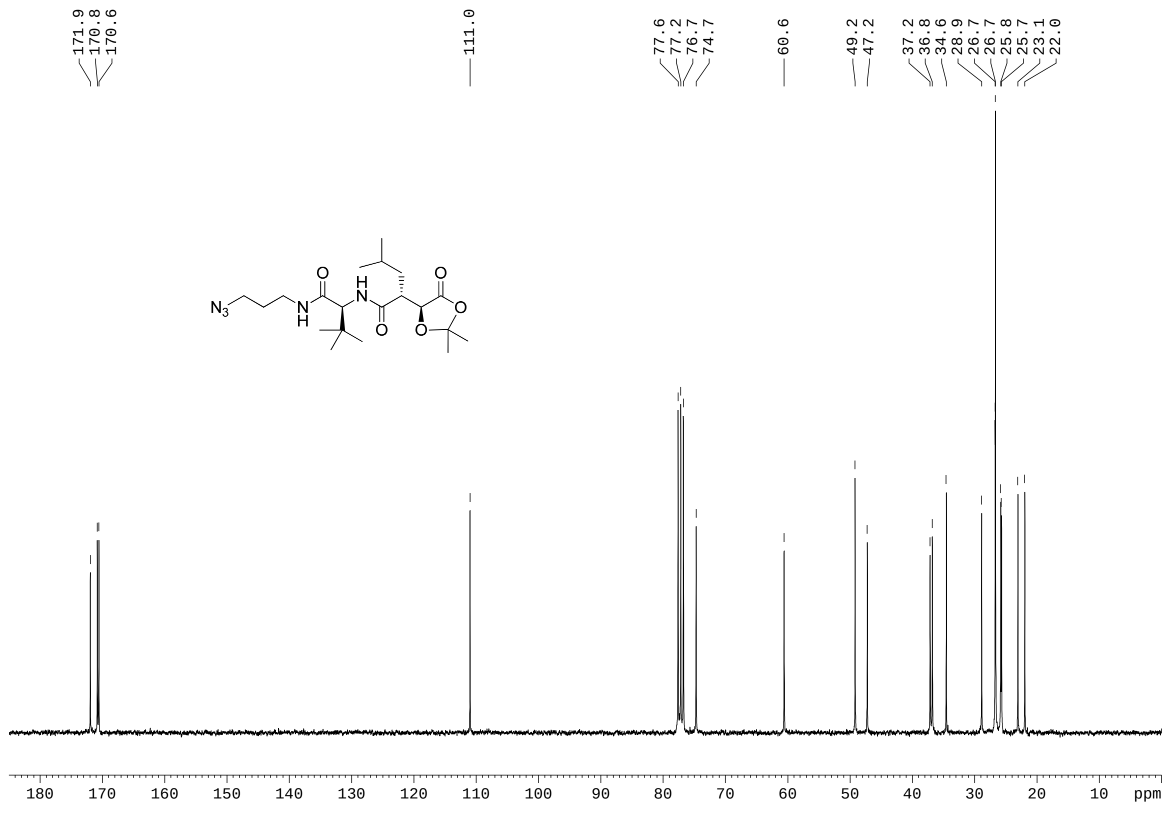

Figure S9. ^{13}C NMR spectrum (75 MHz) of **7** in $CDCl_3$.

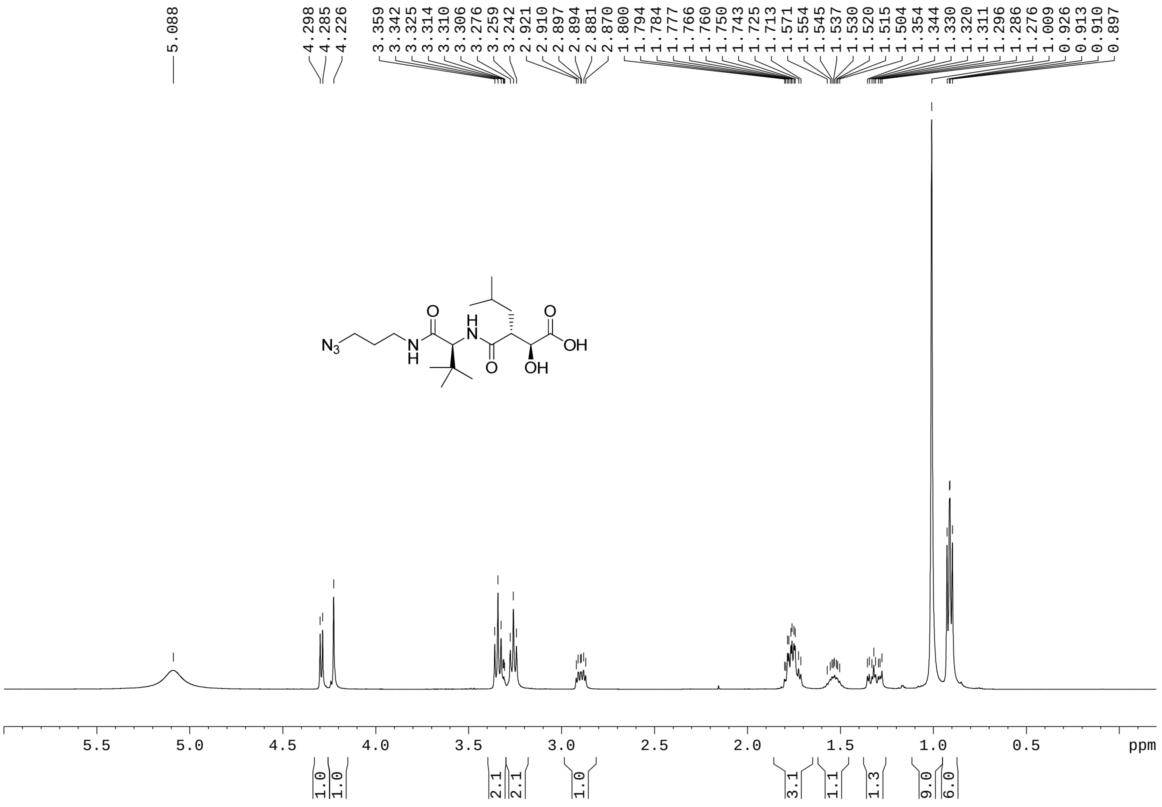

Figure S10. ^{1}H NMR spectrum (400 MHz) of **8** in CD_3OD.

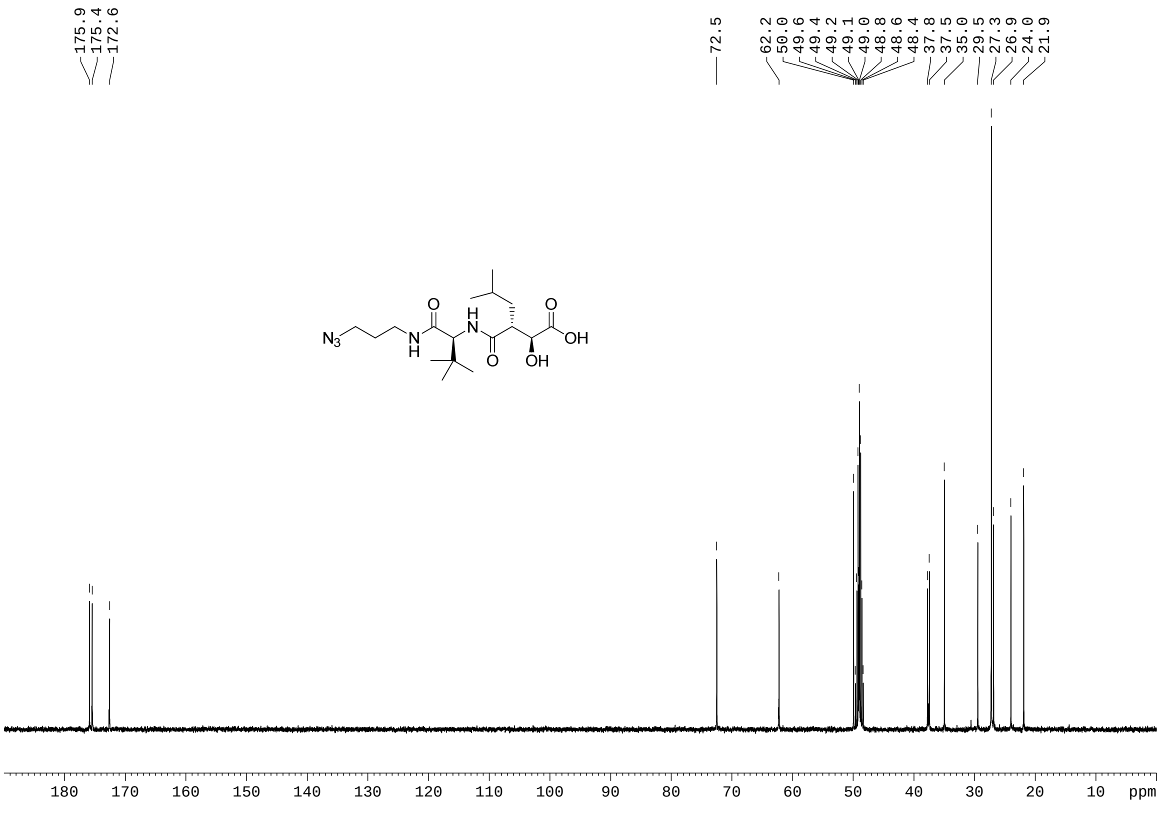

Figure S11. ^{13}C NMR spectrum (100 MHz) of **8** in CD_3OD.

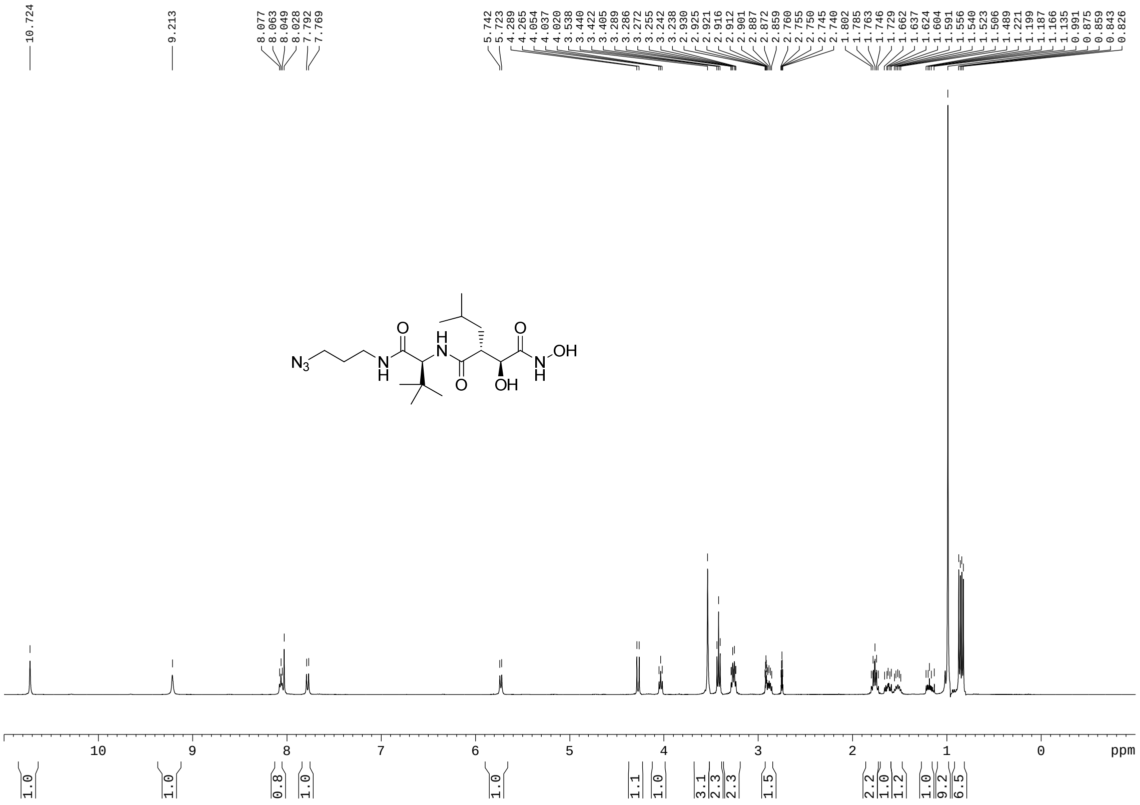

Figure S12. ^{1}H NMR spectrum (400 MHz) of **9** in $DCON(CD_3)_2$.

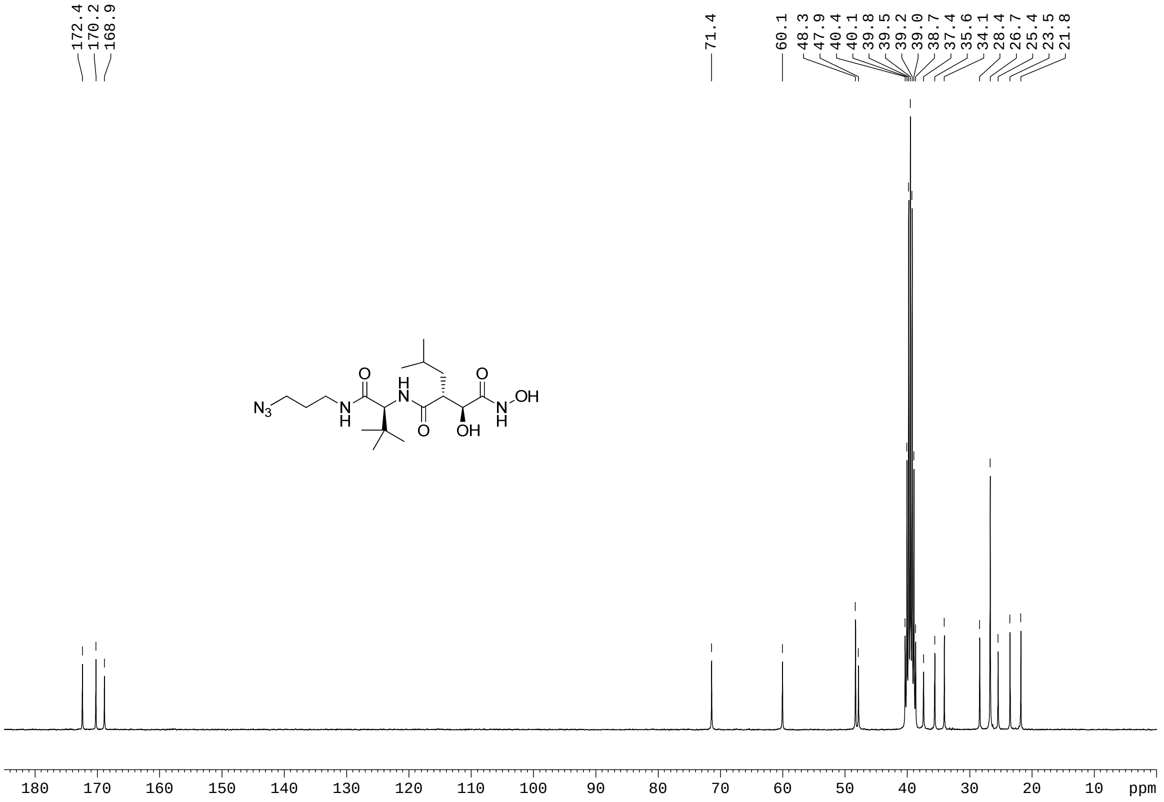

Figure S13. ^{13}C NMR spectrum (75 MHz) of **9** in $(CD_3)_2SO$.

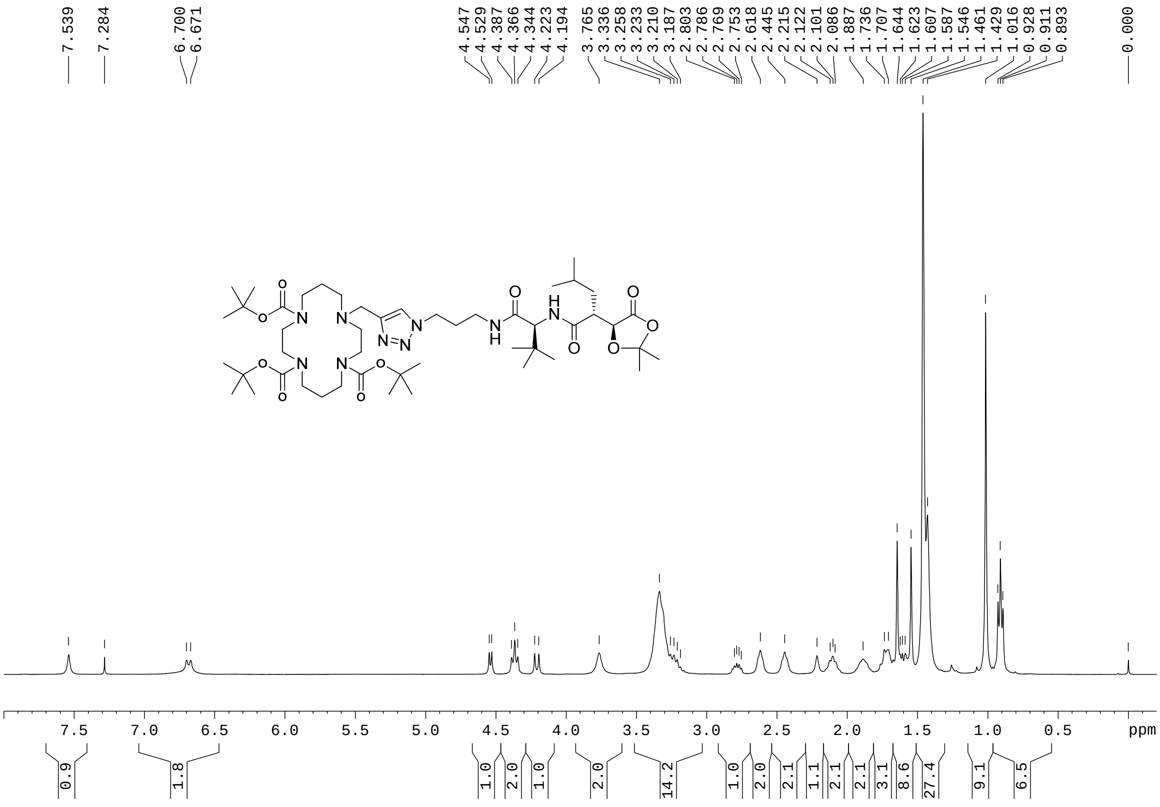

Figure S14. ^{1}H NMR spectrum (300 MHz) of **10** in $CDCl_3$.

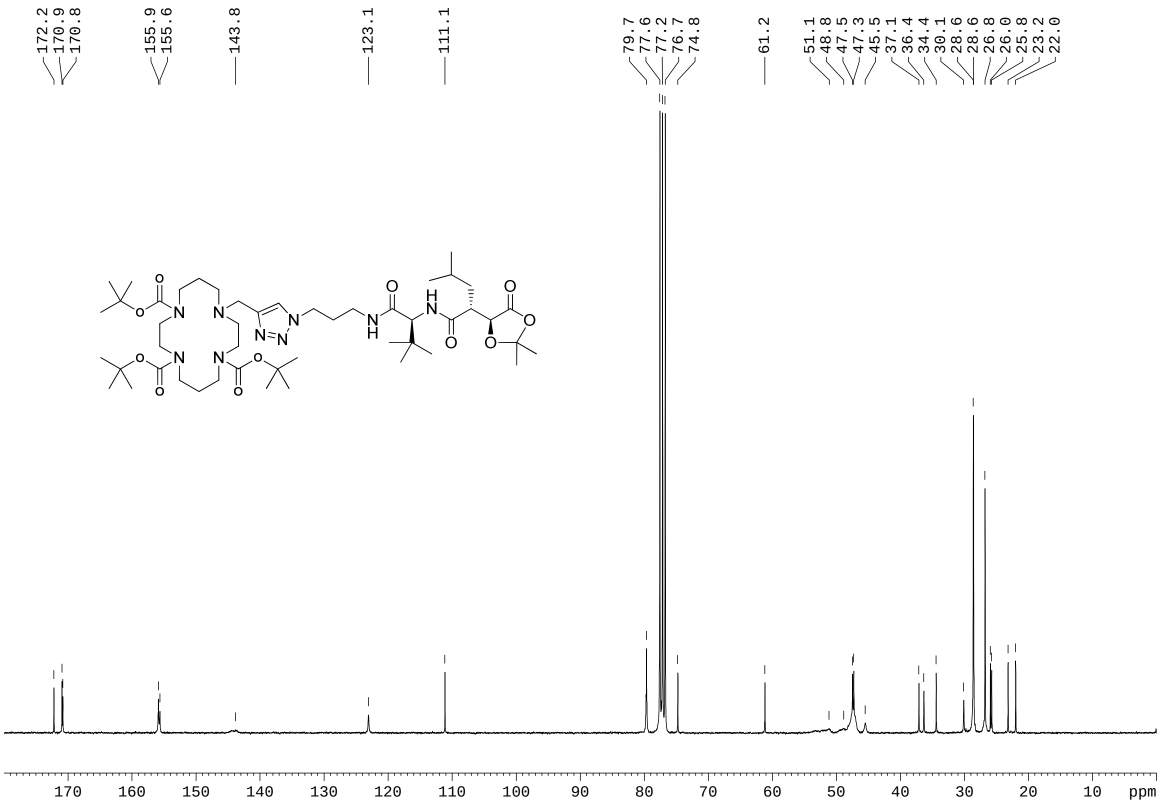

Figure S15. ^{13}C NMR spectrum (75 MHz) of **10** in $CDCl_3$.

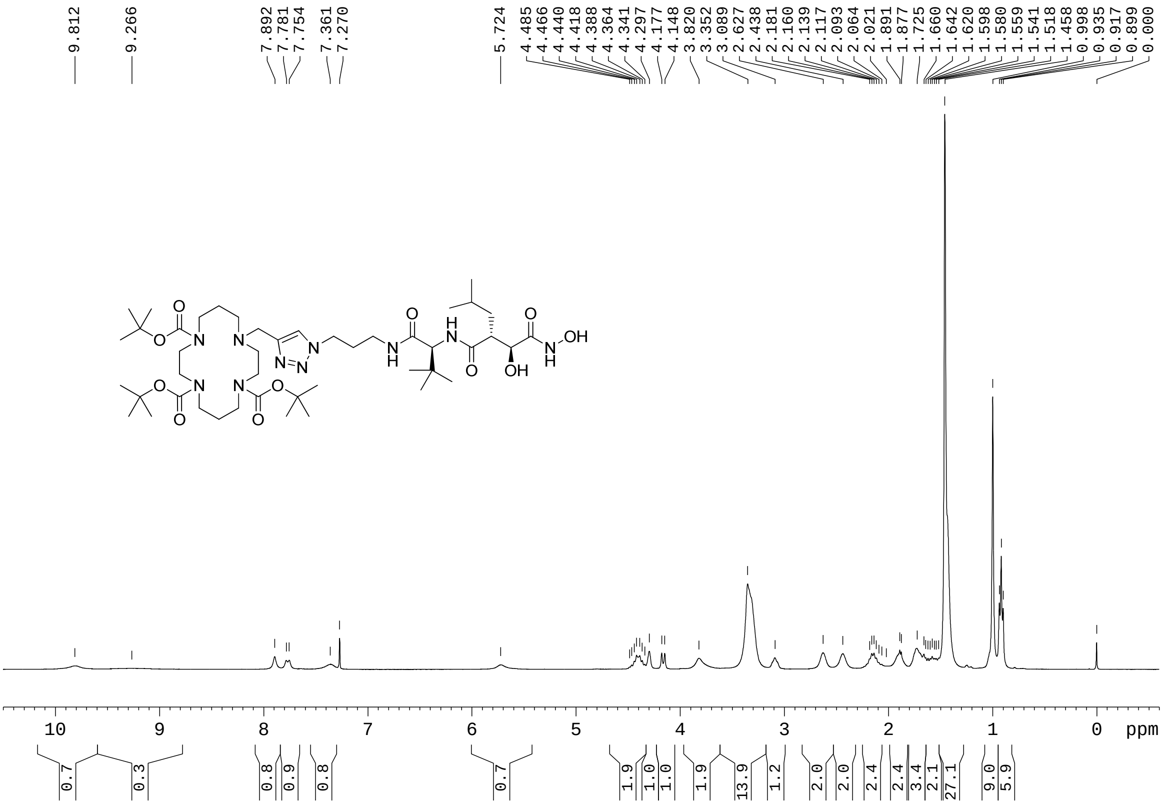

Figure S16. ^{1}H NMR spectrum (300 MHz) of **11** in $CDCl_3$.

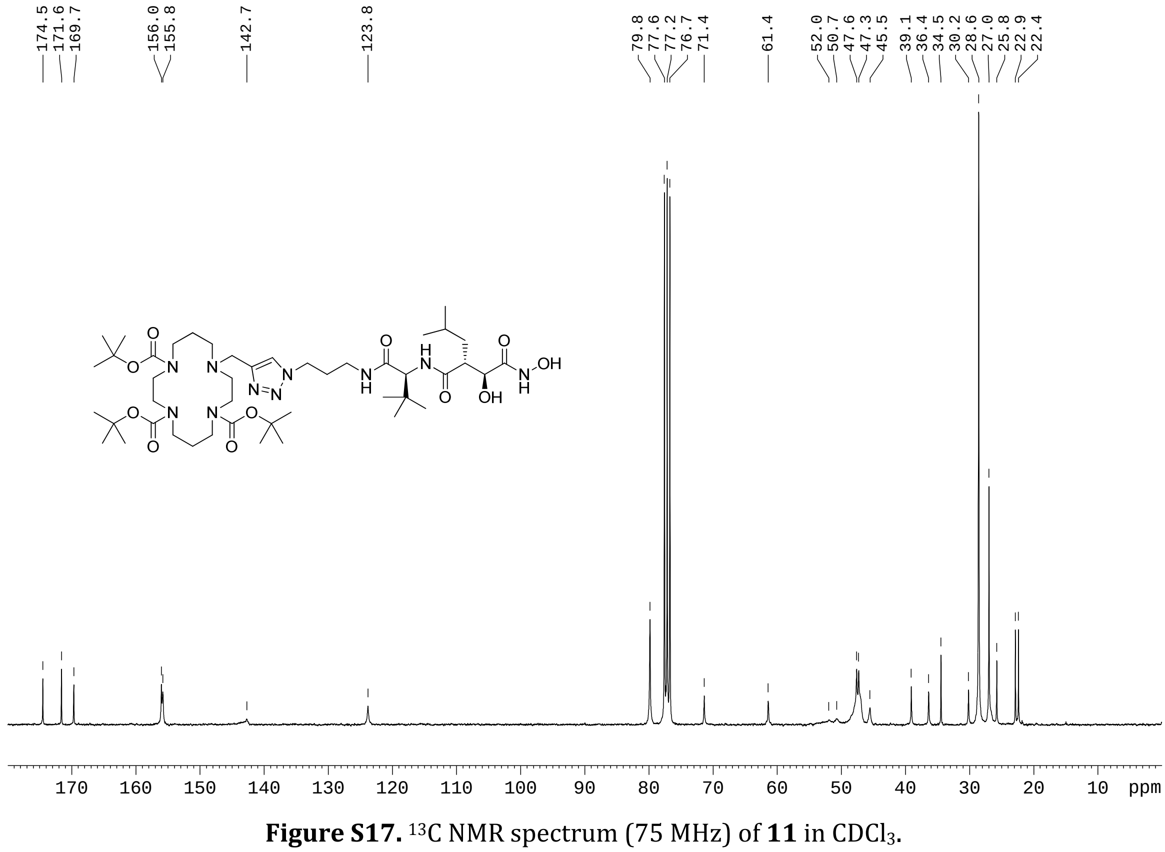

Figure S17. ^{13}C NMR spectrum (75 MHz) of **11** in $CDCl_3$.

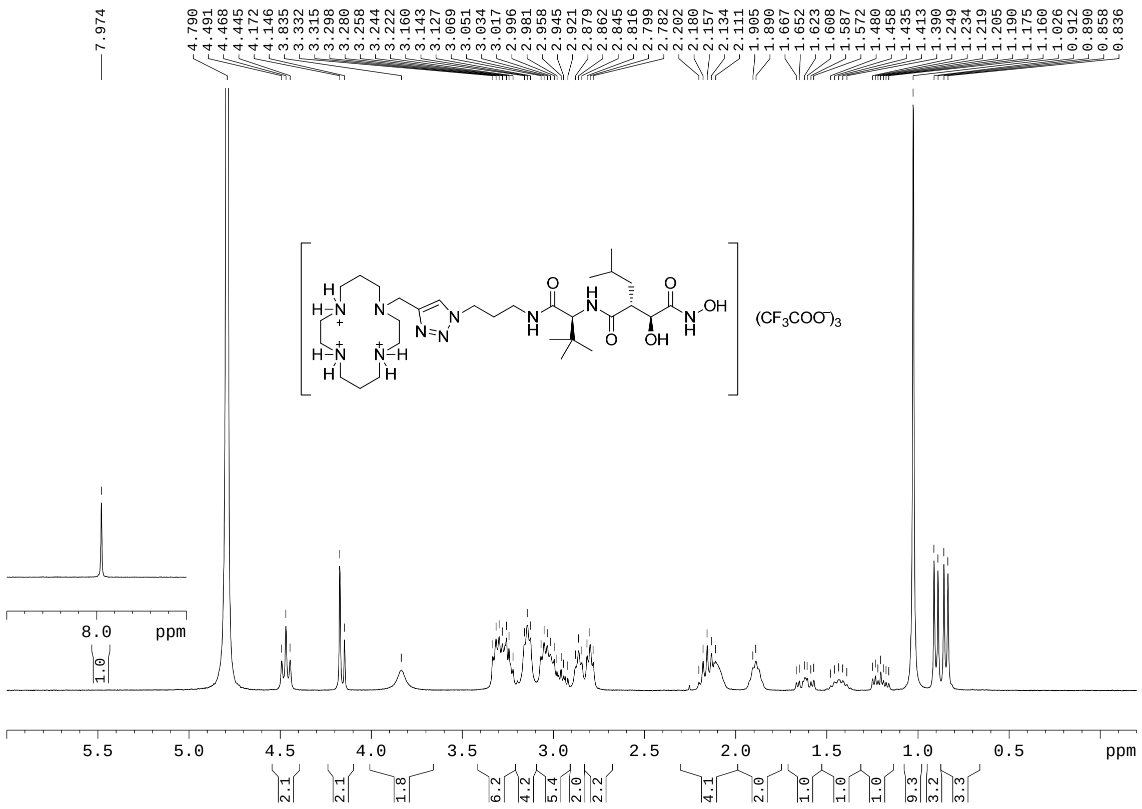

Figure S18. ^{1}H NMR spectrum (300 MHz) of **12** in D_2O.

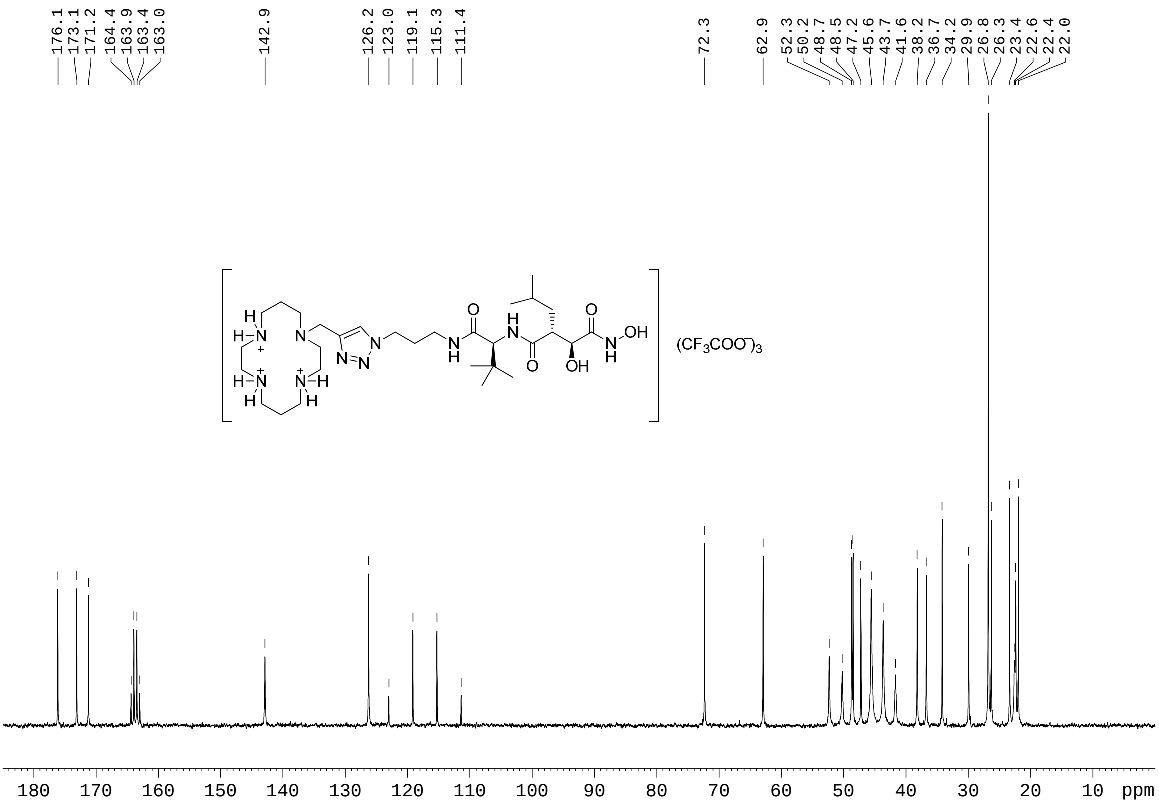

Figure S19. ^{13}C NMR spectrum (75 MHz) of **12** in D_2O.

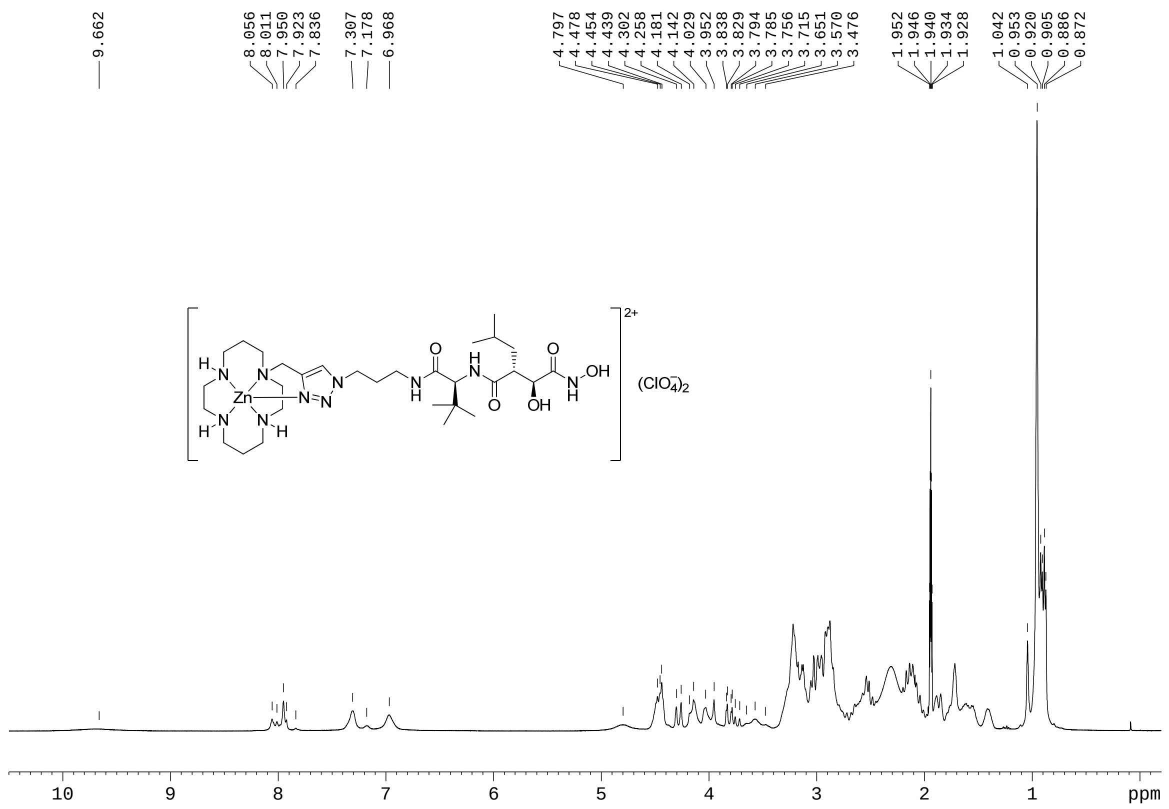

Figure S20. ^{1}H NMR spectrum (400 MHz) of **14** in CD_3CN.

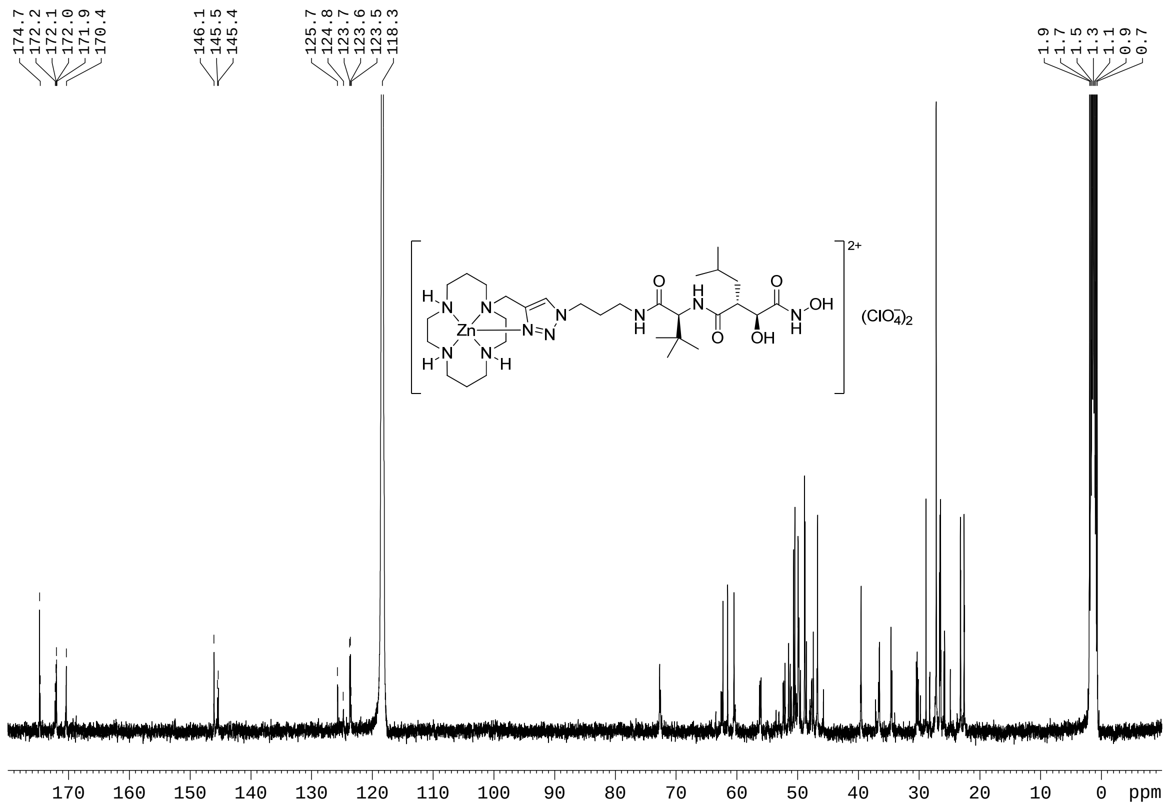

Figure S21. ^{13}C NMR spectrum (100 MHz) of **14** in CD_3CN.
